# Fatty acid transport protein inhibition sensitizes breast and ovarian cancers to oncolytic virus therapy *via* lipid modulation of the tumor microenvironment

**DOI:** 10.3389/fimmu.2023.1099459

**Published:** 2023-03-10

**Authors:** Abera Surendran, Monire Jamalkhah, Joanna Poutou, Rayanna Birtch, Christine Lawson, Jaahnavi Dave, Mathieu J. F. Crupi, Justin Mayer, Victoria Taylor, Julia Petryk, Christiano Tanese de Souza, Neil Moodie, Jacob Lecompte Billingsley, Bradley Austin, Nicole Cormack, Natalie Blamey, Reza Rezaei, Curtis W. McCloskey, Emily E. F. Fekete, Harsimrat K. Birdi, Serge Neault, Taylor R. Jamieson, Brenna Wylie, Sarah Tucker, Taha Azad, Barbara Vanderhyden, Lee-Hwa Tai, John C. Bell, Carolina S. Ilkow

**Affiliations:** ^1^ Ottawa Hospital Research Institute, The Ottawa Hospital, Ottawa, ON, Canada; ^2^ Department of Biochemistry, Microbiology and Immunology, University of Ottawa, Ottawa, ON, Canada; ^3^ Department of Cellular and Molecular Medicine, University of Ottawa, Ottawa, ON, Canada; ^4^ Department of Immunology and Cell Biology, Université de Sherbrooke, Sherbrooke, QC, Canada

**Keywords:** oncolytic viruses, ovarian cancer, breast cancer, adipocytes, fatty acids, viral-based therapeutics, tumor microenvironment

## Abstract

**Introduction:**

Adipocytes in the tumour microenvironment are highly dynamic cells that have an established role in tumour progression, but their impact on anti-cancer therapy resistance is becoming increasingly difficult to overlook.

**Methods:**

We investigated the role of adipose tissue and adipocytes in response to oncolytic virus (OV) therapy in adipose-rich tumours such as breast and ovarian neoplasms.

**Results:**

We show that secreted products in adipocyte-conditioned medium significantly impairs productive virus infection and OV-driven cell death. This effect was not due to the direct neutralization of virions or inhibition of OV entry into host cells. Instead, further investigation of adipocyte secreted factors demonstrated that adipocyte-mediated OV resistance is primarily a lipid-driven phenomenon. When lipid moieties are depleted from the adipocyte-conditioned medium, cancer cells are re-sensitized to OV-mediated destruction. We further demonstrated that blocking fatty acid uptake by cancer cells, in a combinatorial strategy with virotherapy, has clinical translational potential to overcome adipocyte-mediated OV resistance.

**Discussion:**

Our findings indicate that while adipocyte secreted factors can impede OV infection, the impairment of OV treatment efficacy can be overcome by modulating lipid flux in the tumour milieu.

## Introduction

The complex tumor microenvironment (TME) consists of a collection of malignant cell types as well as infiltrating and resident cells (e.g., fibroblasts, adipocytes, vascular endothelial cells, and immune cells), secreted factors, and extracellular matrix (1). Due to recent advancements in our understanding of tumor biology, it is well known that the TME plays a critical role in cancer progression; however, there is increasing evidence to suggest that many stromal elements can also significantly modulate the therapeutic effects of cancer drugs (2). Among the TME players, cancer-associated adipocytes are an underappreciated driver of both tumor progression and anti-cancer therapy resistance (3, 4).

The composition of the TME varies significantly between tumor types (1). A hallmark feature of breast tumors and metastatic ovarian cancers is the abundant presence of surrounding and infiltrating adipocytes (3, 5–9). Adipocytes in the fatty breast TME play critical roles in promoting tumor progression by stimulating cancer cell motility and invasion (10, 11). Over 80% of women diagnosed with ovarian cancer exhibit metastasis in a large abdominal pad of fat cells, called the omentum (7). Nieman and colleagues eloquently showed that adipocytes promote both homing of ovarian cancer cells to the omentum and tumor growth through adipocyte-secreted interleukins and other factors (8). In addition, adipocytes have a demonstrated role in actively promoting anti-breast and ovarian cancer therapy resistance across a wide range of treatment modalities, including chemotherapy, radiation, targeted therapy, monoclonal antibody therapy, and even immunotherapies (4, 6, 12–15). Recent studies revealed that the transfer of bioactive molecules from the omental microenvironment decreased chemotherapy-induced apoptosis of ovarian tumor cells (16, 17). In the context of breast cancer, surrounding adipocytes promote neoplastic cell resistance to the anti-human epidermal growth factor receptor 2 protein antibody Trastuzumab (3), and expression of programmed cell death-ligand 1 (PD-L1) in mammary adipocytes attenuates anti-tumor immunity (6). The mechanisms of adipocyte-driven resistance are diverse, and this is strongly reflective of the complex ways in which adipocytes can communicate with cancer cells and condition the TME.

Cancer-lysing or oncolytic viruses (OVs) are a unique class of cancer immunotherapeutic drugs (18). Infected cancer cells eventually succumb to oncolysis, which facilitates the spread of the virus in the TME to further infection, tumor debulking, and even abscopal effects due to a systemic response despite a localized treatment (19). Moreover, tumor lysis and the release of tumor-associated antigens act as *in situ* vaccination in the TME and contribute to generating a robust host-mediated anti-tumor immune response to wake up immunologically inert or ‘cold’ tumors to become pro-inflammatory or ‘hot’ (19). Several decades of pre-clinical optimization and clinical testing preceded the regulatory approval of different virotherapeutics for cancer treatment. An oncolytic picornavirus, named Rigvir^®^, was the first platform that achieved approval in 2004 for treating people with advance melanoma in Latvia (20). Shortly after, China approved Oncorine (H101), an attenuated adenoviral vector for the treatment of head and neck cancer in combination with chemotherapy (21). Nearly a decade later, the FDA and the EMA approved T-VEC (Imlygic^®^), a genetically modified herpes simplex virus-1 (HSV-1) for treating unresectable melanoma (22). Since then, many other viruses, including oncolytic vesicular stomatitis virus (VSVΔ51) (23–25), vaccinia virus (VACV) (26, 27), and measles virus (MeV) (28) have been exploited as cancer-killing agents and evaluated in pre-clinical and clinical studies globally.

The hallmarks of cancer also act as cellular properties that confer selectivity for OV therapy (29); however, successful tumor colonization by OVs is partly governed by the tumor ecosystem and crosstalk between its cellular compartments. As obligate intracellular parasites, viruses, including OVs, seek a niche favorable for their growth and spread. Given the enrichment of adipocyte-derived fatty acid species in the adipose-rich TME, we speculated about the role of adipocyte-derived lipids in the context of OV infection. Numerous studies evaluating the effect of fatty acids on virus infection have demonstrated pleiotropic effects (30–33). To determine if a lipid-rich niche impacts the activity of clinically staged OVs, we evaluated tumors in adipose-rich TMEs *in vivo* or in cancer cells cultured with adipocyte-secreted factors *in vitro*. We found that tumors in a fatty niche were more resistant to OV infection compared to tumors grown in less fatty tissues. When lipid-derived constituents were depleted from the adipocyte-conditioned medium (ACM), OV infection and OV-mediated cell death were re-instated. These findings led us to investigate the effects of depleting or inhibiting specific fatty acid transport proteins (FATPs) on OV infection and OV-mediated killing in the presence of adipocyte-secreted factors. The combination of virotherapy with a FATP inhibitor improved OV infection *in vitro* and enhanced survival in models of syngeneic fat-pad localized breast cancer or intraperitoneal ovarian cancer. Our findings show, for the first time, that fatty acid blockade in lipid-rich TMEs can sensitize resistant tumors to oncolytic virotherapy.

## Results

### A fat-rich microenvironment impedes productive OV infection

**Figure 1:**
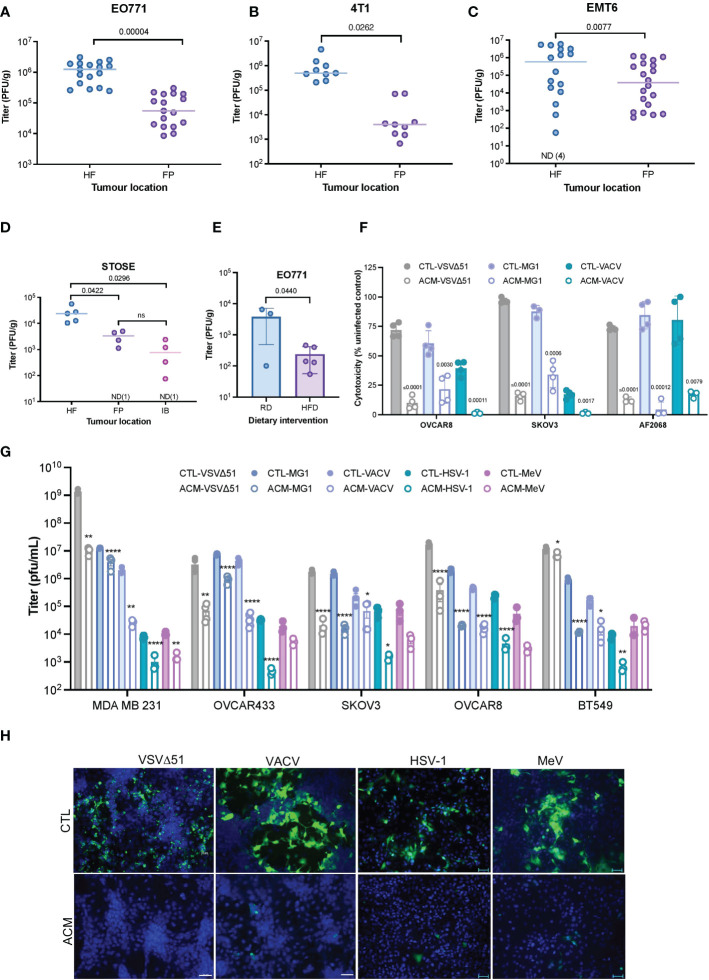
A Fatty TME correlates with OV resistance. **(A–C)** Syngeneic EO771 **(A)**, 4T1 **(B)**, and EMT6 **(C)** breast tumors were seeded in the fat-pad (FP) or subcutaneously in the hind flank (HF) of Balb/c or C57BL/6 mice. Tumors were intratumorally treated with VSVΔ51 [1.5E8 plaque forming units (PFU)/tumor] and collected after 48 hours for quantification of infectious particles. ND, non-detected. Two-tailed, unpaired t-test. **(D)** STOSE cells were seeded in the HF, FP or intrabursally (IB) in FVB/N mice and treated with Maraba MG1 (1E7 PFU/tumor). Tumors were harvested 72 hours post-infection (hpi) for virus quantification by plaque assay. ND, non-detected. Lines indicates the median. One-way ANOVA, Tukey’s multiple comparison test. **(E)** Following a period of high-fat or regular diet feeding of C57BL/6 mice, EO771 cells were seeded in the FP. Tumors were intratumorally treated with Maraba MG1 (5E8 PFU/tumor) and harvested for quantification by plaque assay 48 hpi. Two-tailed, unpaired t-test. **(F)** Relative percentage (% compared to uninfected cells) of VSVΔ51-induced cytotoxicity in OVCAR8, SKOV3, and a primary ovarian cancer cell culture (AF2068) cultured in a regular growth medium (CTL) or a human breast adipocyte-conditioned medium (ACM) for 16 hours prior to infection with indicated OVs (48 hours, MOI 0.1). Results are displayed as mean ± SEM of four biological replicates. Two-tailed, unpaired t-test for each CTL and ACM pair. **(G)** Oncolytic virus titers from an OV-infected panel of breast and ovarian cancer cell lines [OVCAR8, MDA MB 231 and SKOV3 (MOI 0.1, 48 hpi), and OVCAR433 and BT549 (MOI 1, 48 hpi)] cultured in a regular growth medium or ACM were quantified by plaque assay. Data indicate the mean ± SEM of 3 to 5 biological replicates. Two-tailed, unpaired t-test for each CTL vs ACM pair. *p <0.05, **p<0.01, *p<0.05, **p<0.01, ****p<0.0001. **(H)** Representative immunofluorescence microscopy images of OV-infected (VSVΔ51 MOI 0.1 24 hpi, VACV MOI 0.1 48 hpi, HSV-1 MOI 0.1, 48 hpi, MeV MOI 1, 48 hpi) OVCAR8 cells are shown (VSVΔ51 and VACV, scale bar 20μm; HSV-1 and MeV scale bar represents 50μm). ns, non-significant.

Tumor cells can interact with neighboring adipocytes, and this crosstalk appears to reduce the efficacy of certain anti-cancer drugs (34–37). To explore the effect of tumor localization on responses to OV infection *in vivo*, we implanted mouse breast tumors in the mouse hind flank (HF) or mammary fat pad (FP) and then treated them intratumorally with VSVΔ51. In all three syngeneic breast tumor models evaluated (EO771, 4T1, EMT6), tumors seeded near the adipocyte-rich mammary fat pad were significantly less infected than their HF counterparts (**Figures 1A–C**). We observed similar OV infection patterns in a spontaneously transformed ovarian surface epithelial (STOSE) model (38, 39). Tumors in the FP or intrabursal (IB) to the ovary were significantly more resistant to OV infection than subcutaneous HF tumors (**Figure 1D**). C57BL/6 mice bearing mammary FP breast tumors were fed either a high-fat diet (HFD) or a regular chow diet (RD) to impact their body weight (**Supplementary Figure 1A**) and adipose tissue invasion into the tumor (**Supplementary Figure 1B**). Mice fed with HFD showed decreased intratumoral OV titer in comparison to mice on RD (**Figure 1E**), suggesting that the increased presence of adipocytes in the fatty TME impairs virus replication in the tumor.

To explore potential effects of adipocytes on OV infection, we assessed the role of adipocyte-secreted factors in promoting or inhibiting viral replication. A representative panel of breast and ovarian cancer cell lines, including an ascites-derived ovarian cancer cell culture (AF2068) were exposed to adipocyte-conditioned medium [(ACM), **Supplementary Figure 1C**] and infected with clinically relevant OVs (19), including oncolytic VSVΔ51 (23, 24), Maraba MG1 (40), VACV (26, 27), MeV (28) and HSV-1 (22). While ACM treatment did not significantly impact cell proliferation in our experimental settings (**Supplementary Figure 1D**), we observed decreased OV-induced cytotoxicity (**Figure 1F**) and virus replication in the presence of ACM compared to control medium (CTL) (**Figure 1G**) or pre-adipocyte conditioned medium-receiving cells (**Supplementary Figure 1E**). The stark nature of this resistant effect is dose-dependent (**Supplementary Figure 1F**) and apparent in the immunofluorescence microscopy images of OV-infected ovarian and breast cancer cells, as shown in **Figure 1H** and **Supplementary Figure 1G**. Furthermore, ACM from an alternative adipose depot, such as human visceral adipocytes (**Supplementary Figure 2A–C**), or different species [(i.e., mouse adipocytes) **Supplementary Figure 2D, F**] also inhibited OV infection as observed with human breast adipocytes. These data suggest that one or more secreted products of human or murine adipocyte lineage contribute to OV resistance.

### ACM-mediated impairment to OV replication occurs post-virus entry

**Figure 2:**
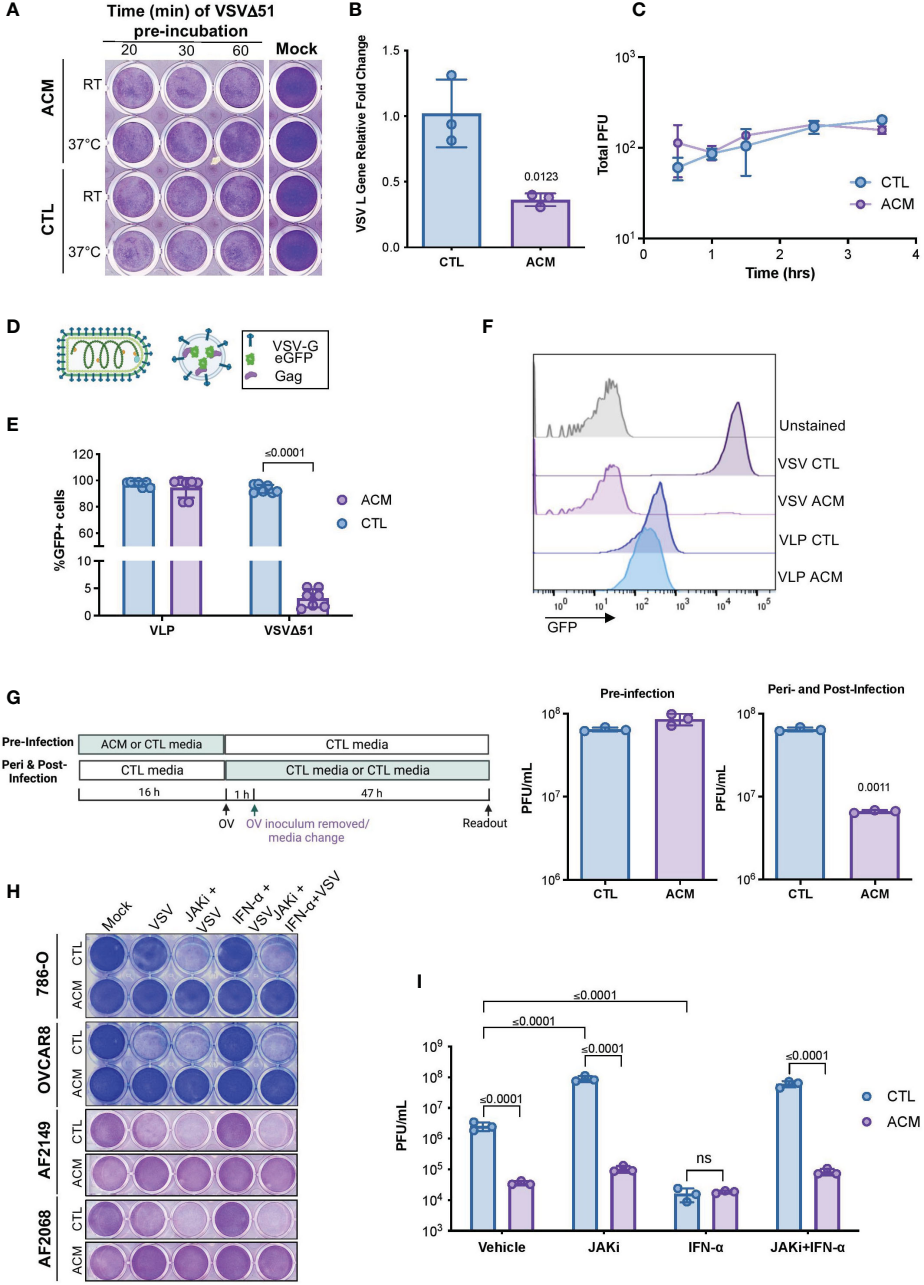
ACM-mediated OV resistance occurs post-virus entry and is a Type I IFN-independent phenomenon. **(A)** Cytotoxicity induced by VSVΔ51 inoculums (MOI 1, 48hpi) that were pre-incubated in CTL medium or ACM at room temperature (RT) or 37°C for the indicated lengths of times and then used to infect OVCAR8 cells in CTL medium. **(B)** OVCAR8 cells in CTL medium were infected with MG1ΔG (MOI 3). After 1 hour, the virus inoculum was removed and changed to CTL medium or ACM. The RNA was extracted at 24 hpi, and the expression of VSV gene L was evaluated by qPCR relative to cellular Rplp0 loading control. Data indicate the mean ± SEM of three biological replicates. Two-tailed, unpaired t-test. **(C)** Cells were incubated in CTL medium or ACM for the indicated amount of time prior to infection with VSVΔ51 (150 infectious particles). Following 1 hour of infection, the virus inoculum was removed and replaced with a semi-solid overlay. After 48, viral titers (in PFU) were quantified and plotted. Lines connect means. Two-way ANOVA, Sidak’s multiple comparison test. **(D)** Cartoon comparing a VSV-eGFP virion and a GFP-loaded VLP pseudotyped with VSV-G. **(E, F)** CTL medium or ACM-receiving cells were infected with GFP-expressing VSVΔ51 (MOI 0.1) or transduced with GFP-loaded VLPs pseudotyped with VSV-G for 24 hours and percentage of GFP positive cells was assessed by flow cytometry. Data indicate the mean ± SEM (n=7). Two-tailed, unpaired t-test for VLP or VSVΔ51-infected samples. **(G)** As summarized in the displayed experimental timeline, OVCAR8 cells were cultured overnight in CTL medium or ACM, and the medium was changed to CTL medium at the time of VSVΔ51 infection. Alternatively, the medium was changed to a CTL medium or ACM at the time of infection. The virus inoculum-containing medium was removed following an hour of infection and replaced with the same medium at the time of infection. Viral titers were quantified by plaque assay. Data indicate the mean ± SEM of 3 biological replicates. Two-tailed, unpaired t-test for each CTL vs ACM pair. **(H, I)** Cell lines or patient ascites cells were cultured in CTL medium or ACM overnight and treated with JAK I inhibitor (1μM), IFN-α (200 U/mL), or both prior to infection with VSVΔ51 (MOI 1) for 48 hours. Cytotoxicity assay was performed to evaluate virus-mediated cell death **(H)**, and the supernatant was collected for quantification of infectious particles by plaque assay **(I)**. Data indicate the mean ± SEM of 3 biological replicates. Two-way ANOVA, Tukey’s multiple comparison test on log 10 transformed data.

To better understand the phenomenon of adipocyte-driven OV resistance, we sought to examine various steps of the virus replication cycle. A virus must first attach to and penetrate a host cell to make copies of itself to generate new virus particles that can then spread to neighboring cells (19). Thus, we first evaluated whether the pre-treatment of virions in ACM was sufficient to neutralize the infectious particles and thus impair their attachment and internalization in cancer cells. Our data revealed that a direct virus-neutralizing agent is unlikely to be the driver of ACM-mediated OV inhibition. Pre-incubation of virus particles with ACM did not compromise OV infection or virus-induce cell death (**Figure 2A**). Furthermore, we conducted a modified plaque formation assay in which cancer cells were infected in the presence of ACM for varying lengths of time. Next, the virus inoculum-containing medium was replaced with a semi-solid medium that permitted the formation of viral plaques. While OV infection in ACM conditions leads to a significant decrease in viral transcripts (**Figure 2B**), the plaque formation experiment revealed that exposure to ACM solely at the time of infection or early-on in the infectious cycle (0-4 hrs) does not significantly impair OV infection (**Figure 2C**). To further investigate the role of ACM on virus entry, we employed virus-like particles (VLPs). VLPs, also known as viral “empty shells”, can mimic the structural properties of native viruses without the capacity for replication since they do not carry a viral genome. We employed a retrovirus-derived VLP platform (41) containing Gag proteins fused to the green fluorescent protein (Gag-GFP) and pseudotyped with VSV glycoprotein (VSV-G) (**Figure 2D**). In ACM treated cells, the percentage of GFP positive cells following VSV infection was diminished considerably, whereas the percentage of GFP positive VLP-transduced cells was not significantly (p= 0.35) altered (**Figures 2E, F**, **Supplementary Figure 3**). Thus, VSV-G-mediated cell entry appeared unhindered in ACM despite a severe impairment to virus replication. A comprehensive evaluation of the effect of the timing of ACM exposure on OV titers corroborated the findings from the VLP studies, suggesting that ACM exposure at later stages of the infection cycle has the most significant influence on mounting OV resistance (**Figure 2G**). Taken together, these findings suggest that adipocyte-secreted factors reduce OV infection at a step after virus entry.

### ACM-mediated OV resistance is not driven by Type I interferon

Adipocytes are endocrine cells that secrete a variety of bioactive molecules, including cytokines and adipokines (7, 8, 42). Type I interferons (IFN-I) are often the first line of defense in the innate anti-viral response (29, 43). Given the robust resistance of otherwise OV-sensitive cancer cells in the presence of adipocyte-secreted factors, we first sought to determine the potential role of IFN-I signaling on ACM-mediated OV resistance. IFN-α engages with the cellular interferon-α/β receptor (IFNAR). Topical treatment with IFN-α served as a positive control for engaging anti-viral signaling associated with an IFNAR-mediated interferon signaling cascade. When IFN-α treated cells were infected with the highly IFN-sensitive oncolytic VSVΔ51, little to no infection was observed (**Supplementary Figure 4A**). When cells were treated with both IFN-α and an IFNAR blocking antibody, the cells became vulnerable to VSVΔ51 infection, demonstrating that the concentration range of IFNAR blocking antibody was sufficient to block the binding and engagement of IFNAR ligands. Yet, in cancer cells receiving ACM, the addition of IFNAR blocking antibody did not alleviate resistance to VSVΔ51 infection, suggesting an IFNAR-independent mechanism of OV resistance (**Supplementary Figure 4A**). Like most proteins, IFNs are thermolabile and sensitive to proteolytic treatment (44). We complemented the above findings by evaluating the effects of proteinase K treatment, heat-inactivation, or boiling of ACM, which led to no notable effects on mitigating OV resistance (**Supplementary Figure 4B–G**). Interferons work through activation of the Janus kinase-signal transducer and activator of transcription (JAK-STAT) pathway to activate a plethora of genes that are collectively known as interferon-stimulated genes (29). To further validate that the ability of ACM to induce an anti-viral state is IFN-I independent, we evaluated the effect of blocking downstream IFN signaling using JAK inhibitors. JAK inhibitor-treated ACM-receiving cancer cell lines or patient ascites-derived cell cultures (AF2068 and AF2149) remained firmly resistant to infection despite a sensitizing effect in controls (**Figures 2H, I**). Collectively, these findings suggest that the ACM-driven OV resistance is a type-I IFN-independent phenomenon.

### Adipocyte-derived lipid moieties are the primary driver of OV resistance

**Figure 3:**
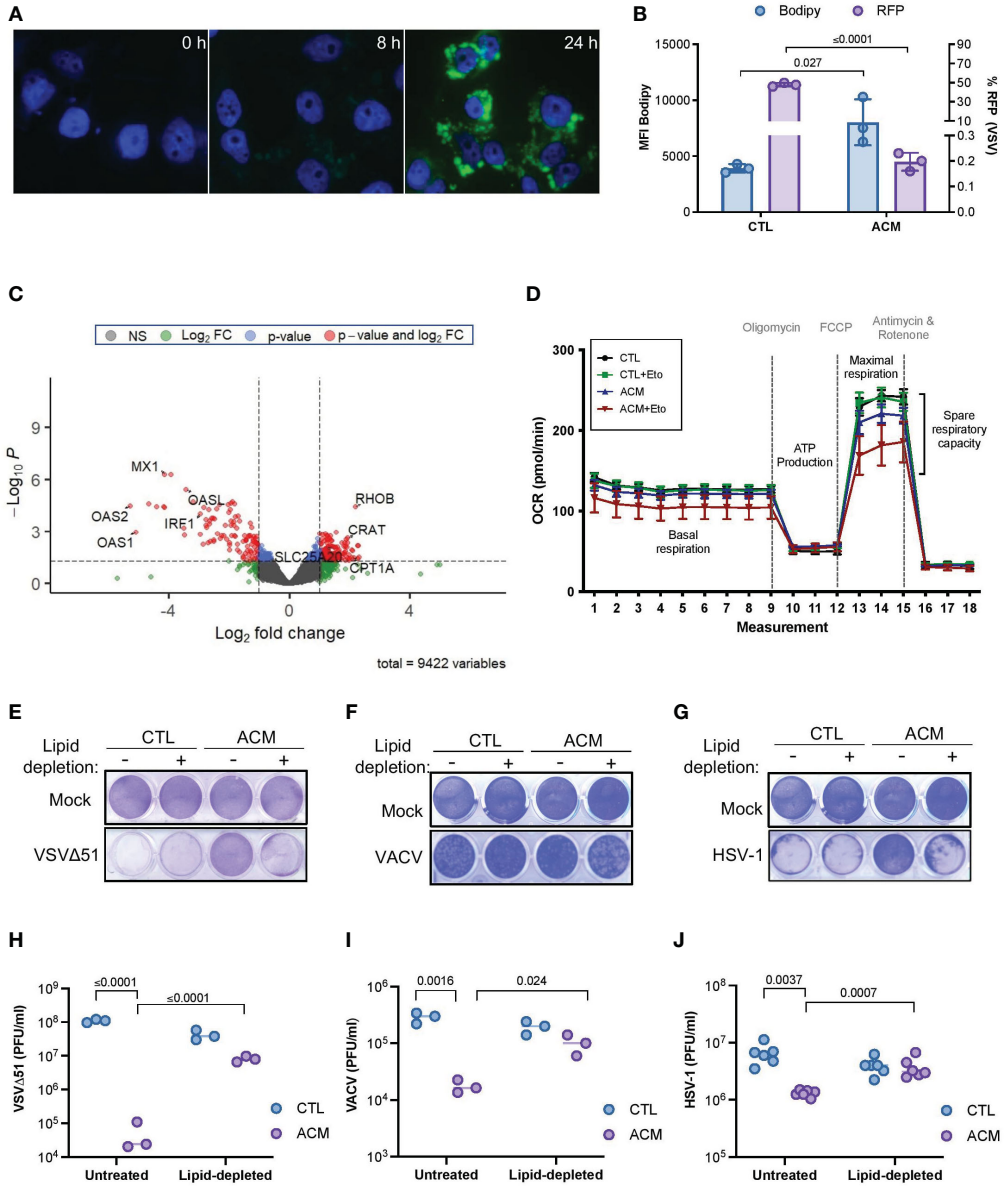
Adipocyte-derived lipid moieties are the key drivers of OV resistance. **(A)** OVCAR8 cells cultured in ACM for the indicated lengths of time and stained with Bodipy to label neutral lipid content. DAPI denotes nuclei staining. **(B)** SKOV3 cells were infected with an RFP-expressing VSVΔ51 (MOI 3, 18 hours) and stained with Bodipy for flow cytometry analysis. Data indicate the mean ± SEM of 3 biological replicates. Two-tailed, unpaired t-test. **(C)** OVCAR8 cells were cultured in ACM or CTL-medium for 18 hours prior to harvesting total RNA for RNA sequencing analysis. The data was analyzed relative to ACM to visualize the relative changes of gene expression in ACM from two biological replicates. Y-axis dotted line denotes a p-value cut-off of 0.05. X-axis dotted lines denote a 2-fold change cut off. **(D)** Oxygen consumption rate (OCR) in OVCAR8 cells cultured in a control medium or ACM was assessed using Agilent Seahorse technology. Oligomycin, trifluoromethoxy carbonylcyanide phenylhydrazone (FCCP), and antimycin and rotenone treatment at designated intervals. Acute treatment with Etomoxir (Eto) was assessed in parallel. **(E–J)** Lipid-depleted control or ACM medium was added to OVCAR8 cells for overnight culturing and infected with VSVΔ51 (MOI 0.1) or VACV (MOI 1) or HSV-1 (MOI 0.1). Virus-induced cytotoxicity is shown by crystal violet staining of attached cells **(E–G)**. The number of infectious particles released by cancer cells cultured in the indicated conditions after 48 hours of infection was assessed by plaque assay **(H–J)**. Data indicate the mean ± SEM of 3 biological replicates. Two-way ANOVA, Tukey’s multiple comparison test on log 10 transformed data.

Adipocytes are highly metabolic cells that store lipids and release them as free fatty acids (12). Consistent with previous findings (7, 8), we found that the level of fatty acid in ACM compared to control medium was significantly higher (*p*=0.004) (**Supplementary Figure 5A**), and cancer cells grown in the presence of ACM accumulated intracellular lipids over time (**Figures 3A, B**). Moreover, we observed an inverse correlation between intracellular lipid accumulation and OV infection (**Figure 3B**). Close examination of the transcriptome of ACM-treated cancer cells also revealed an increase in the expression of critical genes involved in fatty acid metabolism, including carnitine o-acyl transferase (CRAT), carnitine acylcarnitine translocase (SLC25A2O) and carnitine palmitoyl transferase I (CPT1), a critical mitochondrial enzyme involved in the β-oxidation of fatty acids (45) (**Figure 3C** and **Supplemental Figure 5B–D**). We therefore tested whether the accumulation of lipids and changes in the expression of genes involved in fatty acid oxidation contribute to altering the metabolism of cancer cells by assessing mitochondrial metabolic parameters using Agilent Seahorse technology. When etomoxir, a non-reversible inhibitor of CPT1 (45, 46), was added to OVCAR8 ovarian cancer cells, CTL medium-receiving cells showed no notable change in their oxygen consumption rate. In contrast, ACM-receiving cells demonstrated a drastic reduction in the window assessing spare respiratory capacity (**Figure 3D**), suggesting a reliance on CPT1-mediated respiration. These results suggest a metabolic dependency on fatty acids as a fuel source in ACM-receiving cancer cells.

Adipocytes can release large amounts of soluble mediators that can directly or indirectly promote lipid accumulation in cancer cells, thereby contributing to their resistance to oncolytic virotherapy. Accordingly, we next explored whether we could restore OV infection and killing by blocking lipid biosynthesis in cancer cells cultured in ACM. TOFA (5-tetradecyloxy-2-furoic acid) is an allosteric inhibitor of acetyl-CoA carboxylase (ACC), an enzyme critical for long chain fatty acid synthesis (47). Interestingly, we found that when cancer cells were treated with increasing concentrations of TOFA, there was an increase in susceptibility to OV infection and cell death; however, this sensitizing effect was absent in cells that also received adipocyte secreted factors (**Supplementary Figure 6A, B**). These findings may indicate that despite the beneficial effects of combination treatment with TOFA, OV resistance is reinstated when an exogenous source of lipids counteracts the absence of intracellular lipid synthesis. On the contrary, intracellular lipid accumulation decreased when cancer cells received lipid-depleted ACM (**Supplementary Figure 6C**), and this phenomenon correlated with an increase in OV titer and recovery in OV-mediated cytotoxicity (**Figures 3E–J**). Moreover, the inhibitory effect of ACM was similar across two different OV platforms when regular culture medium was supplemented with the fatty acid palmitate or with a chemically defined lipid mixture containing seven fatty acids and cholesterol (**Supplementary Figure 6D–I**). These studies provide functional evidence for the uptake of lipid moieties into receiving cancer cells and strongly suggest that ACM-mediated OV resistance is primarily a lipid-driven phenomenon.

### Blocking FA uptake as a combinatorial strategy to sensitize tumors in a fatty niche to virotherapy

**Figure 4:**
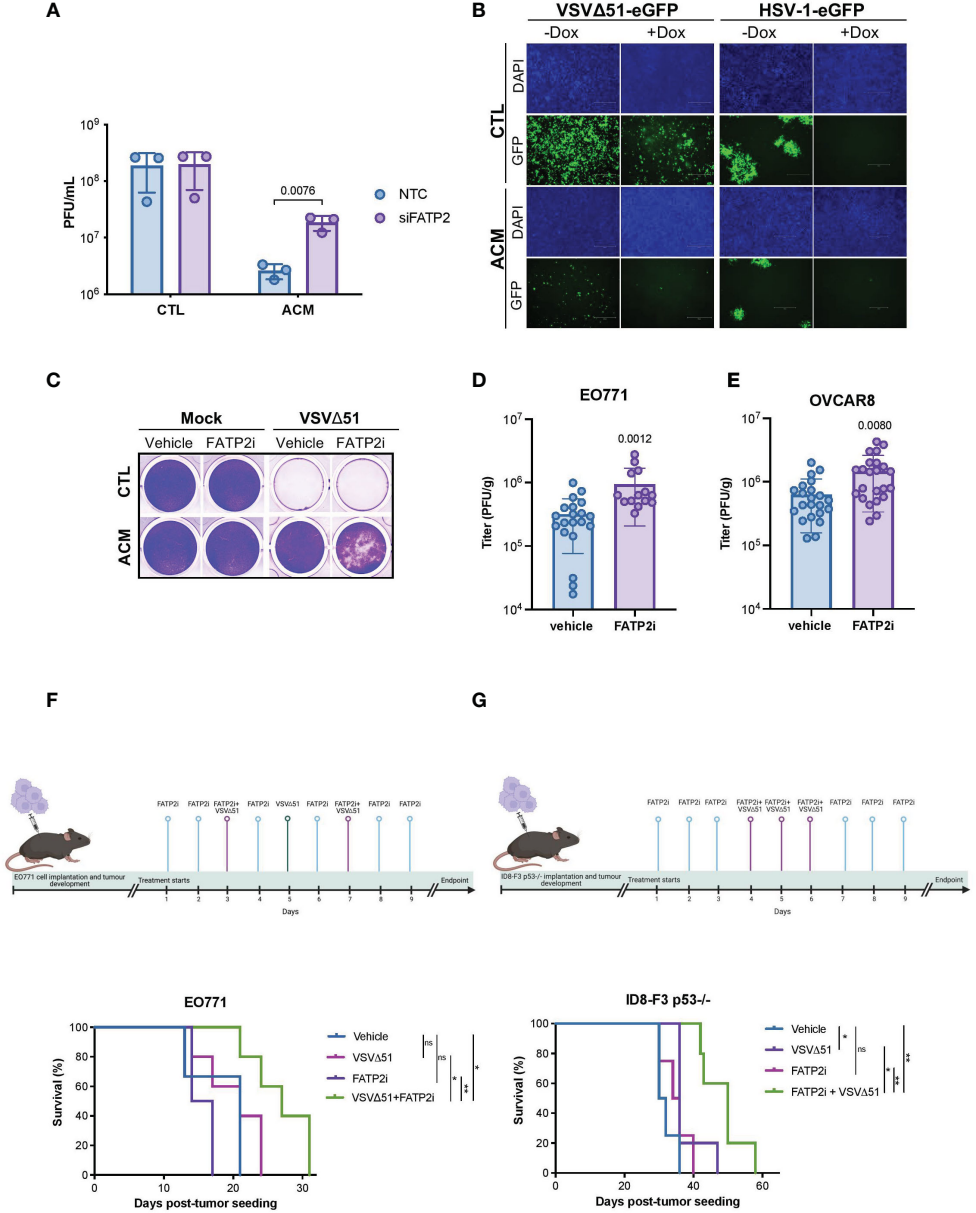
Blocking fatty acid uptake can resensitize cancer cells to OV infection and improve response to OV therapy. **(A)** OVCAR8 cells were transfected with siRNAs targeting *FATP2* (*SLC27A2*) for 16 hours and then infected with VSVΔ51 (MOI 0.05) for an hour. The virus inoculum was removed and replaced with CTL medium or ACM. The supernatants were titrated by plaque assay 48 hpi. Data indicate the mean ± SEM (n=3). Two-way ANOVA, Bonferroni’s multiple comparisons test on log 10 transformed data. **(B)** Representative images of doxycycline (Dox) inducible FATP2 expressing OVCAR8 cells cultured in ACM or CTL medium and infected with VSVΔ51 (MOI 0.01, 48 hpi) or oncolytic HSV-1 (MOI 0.01, 72 hpi) are shown. **(C)** OVCAR8 cells cultured in CTL medium or ACM and treated with vehicle control or a FATP2i (Lipofermata 0.24 μM) were infected with VSVΔ51 (MOI 0.1) and stained with crystal violet (96 hpi). **(D, E)** A single dose of VSVΔ51 was delivered IT into FP E0771 (n=14-20 per group) or OVCAR8 (n=22 per group) tumors. After 48 hours post-delivery, virus titers were quantified. Data represent mean values ± SEM. Unpaired two-tailed t-test. **(F)** Timeline for VSVΔ51 and FATP2i treatment of EO771 bearing mice and Kaplan Meier survival analysis of EO771 fat-pad tumor bearing C57bl/6 mice received vehicle (n=3), FATP2i (3mg/kg) (n=4), VSVΔ51 (1E8 PFU) (n=5) or both FATP2i and VSVΔ51 (n=5) by intratumoral delivery as indicated in the experimental timeline. **(G)** ID8-F3 p53 -/- intraperitoneal (IP) tumor bearing C57bl/6 mice received vehicle (n=4), FATP2i (3mg/kg) (n=4), VSVΔ51 (3E8 PFU) (n=5) or both FATP2i and VSVΔ51 (n=5) by IP delivery and assessed for survival as shown in the timeline. Log-rank (Mantel-Cox) test, *p <0.05, **p <0.01. ns, non-significant.

To develop a strategy that can improve oncolytic virotherapy in adipocyte-rich TMEs, we sought to investigate the effect of blocking the uptake of lipids by cancer cells. Numerous proteins have been implicated in transporting fatty acids, including fatty acid translocase/CD36, caveolin-1, and fatty acid transport proteins (FATP1-6) (48, 49). FATPs are one of the most heavily studied families of proteins involved in the transport of fatty acids. However, of the FATP family members, only FATP1, 2, and 4 have been shown to directly participate in fatty acid transport (50, 51). Thus, we evaluated the effect of FATP1, 2, and 4 downregulation (**Supplementary Figure 7A**) on OV infection of cancer cells. We found that while FATP1 or FATP4 knockdown had no apparent effect on bolstering OV infection (**Supplementary Figure 7B**); FATP2 downregulation noticeably increased OV infection in OVCAR8 cells cultured in ACM (**Figure 4A**, **Supplementary Figure 7B**). Alternatively, when we overexpressed FATP2 in OVCAR8 cells using a tetracycline-inducible system (**Supplementary Figure 7C**), OV infection was reduced in both conditions receiving control medium or ACM (**Figure 4B**). To determine whether adipocyte-secreted lipid molecules and their uptake in cancer cells *via* FATP2 are responsible for the observed OV resistance, we treated FATP2-mediated fatty acid accumulation with a small molecule inhibitor. Lipofermata is a widely studied FATP2 inhibitor (FATP2i) that was identified in a yeast system expressing human FATP2 and has been demonstrated to inhibit FATP2 in cell lines and animal models (52, 53). We found that Lipofermata recapitulated the phenomenon observed in the FATP2 knockdown experiments. While Lipofermata did not impact the viability of OVCAR8 cancer cells (**Supplementary Figure 7D**), we observed that Lipofermanta-treated cells were more susceptible to OV infection and killing in the presence of ACM than the vehicle control-treated counterparts (**Figure 4C** and **Supplementary Figure 7E**).

Next, we examined whether FATP2 inhibition can improve the therapeutic efficacy of an OV in pre-clinical breast and ovarian cancer models. Similar to our observations in the *in vitro* studies, evaluation of the intratumoral oncolytic VSVΔ51 titers in syngeneic breast EO771 mammary fat pad-localized tumors revealed a Lipofermata-driven increase in OV replication at the site of the tumor (**Figure 4D**). We found similar results when we used a human ovarian OVCAR8 tumor model engrafted in immunodeficient animals (**Figure 4E**). Moreover, mice bearing breast EO771 orthotopic tumors and receiving a combination treatment of Lipofermata and oncolytic VSVΔ51 showed the best survival advantage compared to either treatment alone (**Figure 4F**). Similarly, in an immunocompetent model of intraperitoneal ovarian cancer (ID8 p53-/-), combination treatment with Lipofermata provided the best survival advantage, while neither treatment alone provided a survival benefit (**Figure 4G**). The aforementioned pre-clinical studies demonstrate that combination treatment with a FATP2i can enhance intratumoral virus growth and the therapeutic benefits of OV therapy in fatty tumor models.

## Discussion

**Figure 5:**
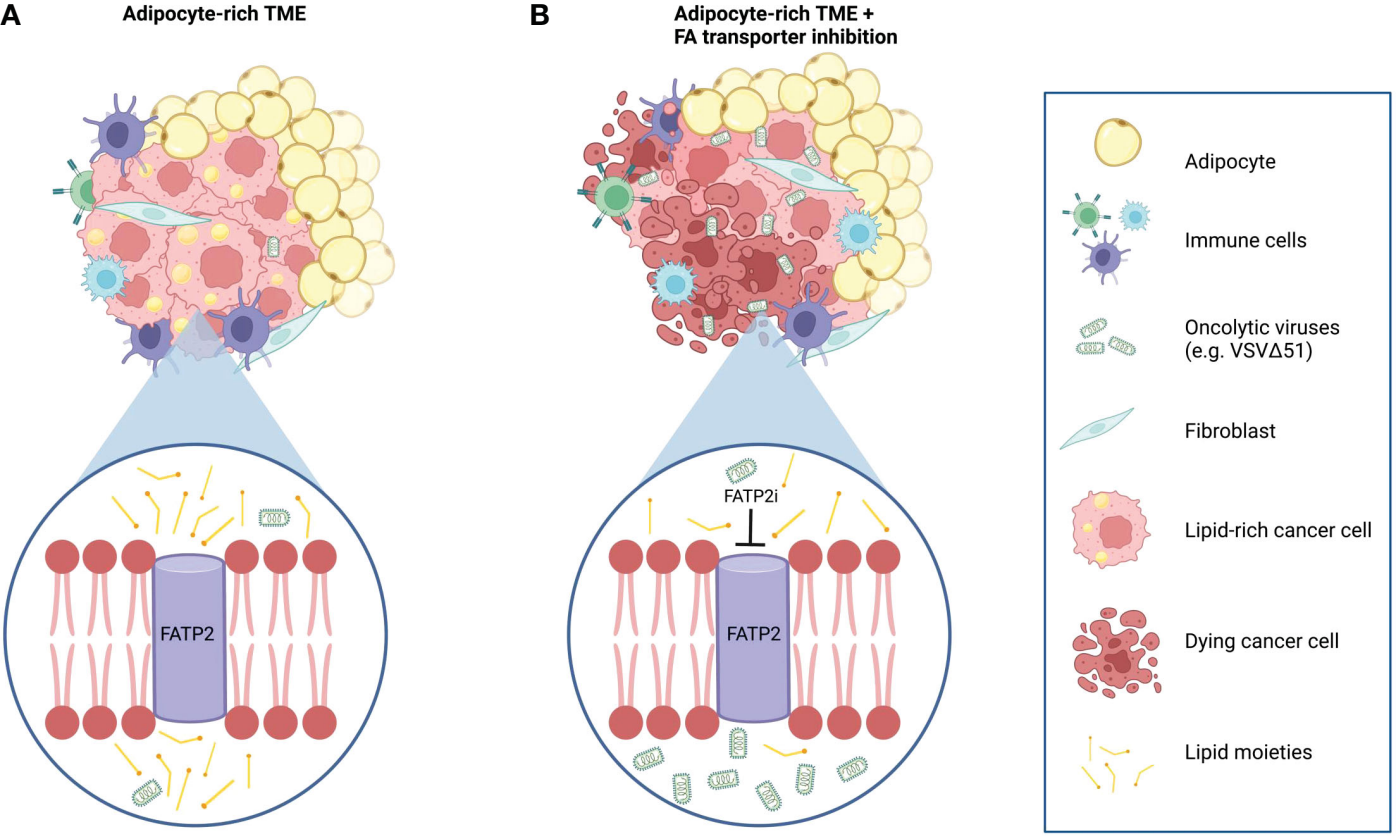
Working model of the effects of adipocyte-derived lipids on OV resistance and the OV sensitizing effects of combination therapy with FATP2 inhibition. **(A)** Tumors in an adipocyte-rich microenvironment accumulate intracellular lipids that lead to a decreased sensitivity to oncolytic virus infection and cancer cell killing. **(B)** Intracellular lipid accumulation can be mitigated by blocking the uptake of exogenous adipocyte-derived lipids by depletion of the lipid-derived constituents in the ACM or by using specific inhibitors to block the activity of fatty acid transporter proteins, such as FATP2. For example, Lipofermanta, a specific FATP2 inhibitor, can re-sensitize cancer cells to OV infection and virotherapy-mediated cell death.

The cellular composition of the TME varies among tumor types creating permissive or hostile niches for oncolytic virus activity. For example, the defining feature of the pancreatic TME is its fibrotic stroma consisting primarily of cancer-associated fibroblasts. We have shown previously that these cells sensitize neoplastic pancreatic cells to OV killing *via* the secretion of FGF-2 (54). Conversely, our *in vitro* and *in vivo* studies presented herein suggest adipose-rich microenvironments as negative modulators of OV therapeutic activity in the breast and ovarian cancer settings. For the first time and as summarized in **Figure 5**, we showed that adipocyte-secreted molecules could severely impair OV replication and impede OV-mediated tumor cell destruction (**Figure 1**, and **Supplementary Figures 1** and **2**). Of note, the inhibitory effect of ACM was observed across viruses from four distinct families (i.e., Rhabdoviridae, Poxviridae, Herpesviridae, and Paramyxoviridae). These oncolytic viruses are genetically diverse and unique in the host receptors they require for cell entry and their modes and sites of replication. The concordant inhibitory effect of ACM on multiple viruses with such wide diversity in their methods of infection and replication suggests that ACM-mediated impediment to OV infection is more likely an indirect effect, for example, through alterations to the host cell, rather than a direct effect on the virions themselves or the virus-receptor interactions. Our data suggest a post-virus entry (**Figures 2A–G** and **Supplementary Figure 3**) and Type I interferon-independent (**Figures 2H, I** and **Supplementary Figure 4**) mechanism of resistance. Viruses are obligate intracellular parasites, and their activity and successful growth depends entirely on the host cell nutrients, energy, and metabolites (55). Future work should focus on deepen our understanding of the potential cellular changes induced by adipocyte-secreted factors, including reprogramming of metabolic pathways, which can impact OV infection and replication in cancer cells.

One of the earliest experiments demonstrating fatty acid-mediated anti-viral effects was observed in breast milk (56). Since then, several studies revealed a potential role of lipids in perturbing infection and replication of various viruses (30–33). The identification of lipid species, including fatty acids, as the primary driver of ACM-mediated OV resistance was a landmark finding in this study (**Figure 3** and **Supplementary Figures 5**, **6**). Our investigations showed that cancer cells treated with ACM accumulated neutral lipids and displayed a resistant state against OVs. We hypothesized that limiting lipid uptake by cancer cells in a fat-rich microenvironment, could override the inhibitory effects of adipocyte-derived metabolites on OV platforms. There are numerous proteins involved in fatty acid transport. Among them, FATPs are one of the most heavily studied. In our study, FATP2 downregulation by siRNAs or FATP2 inhibition with the small molecule Lipofermata improved OV-mediated cell death and OV infection *in vitro* and *in vivo* (**Figure 4** and **Supplementary Figure 7**). Of note, Lipofermata is one of the best-characterized inhibitors of FATP2 with promise for use in the clinic. To date, only a handful of studies have evaluated Lipofermata in the context of cancer (53, 57). These studies showed that the *in vivo* anti-tumor effects of Lipofermata are driven by modification of immune cells in the TME, specifically neutrophils, MDSCs, and indirectly, T-cells (57). While Lipofermata has previously been shown to slow tumor growth, in our studies, Lipofermata monotherapy did not provide significant tumor control or survival benefit. Given that Lipofermata has been demonstrated to have direct anti-cancer activity on cancer cells in a concentration-specific manner *in vitro*, we speculate that the non-toxic concentrations used in our *in vivo* study provided a sufficient fatty acid limiting effect without inducing cellular toxicity that may impair productive OV replication, ultimately supporting the therapeutic benefit of OV combination therapy. Of note, Lipofermata has been shown to be a non-competitive inhibitor for long and very long fatty acids that prevent cellular dysfunction and cell death induced by excessive exposure to fatty acids (52). Here, we have demonstrated that FATP2i improves the therapeutic potential of OV therapy in both immunocompetent and immunodeficient tumor models (**Figure 4**). Our findings build on a growing body of evidence for FATP2 blockade as a therapeutic strategy to overcome anti-cancer therapy resistance (58). However, future work should explore the therapeutic benefits of blocking other modes of fatty acid transport. While downregulation of other fatty acid transporters, such as FATP1 and FATP4, did not improve the sensitivity of ACM-treated cells to OV infection in our models (**Supplementary Figures 7A, B**), there are many other membrane-associated putative fatty acid carriers that display increased expression in cancer cells, including FATPs other than FATP2, the fatty acid translocase CD36 and fatty acid binding proteins (FABPs) (59–61). Notably, CD36 has demonstrated roles in potentiating tumor growth and anti-cancer treatment resistance by facilitating the transport FA substrates in the tumor microenvironment (62–64). Given that CD36 has been implicated in driving both ovarian and breast cancers, it could be an attractive target in CD36-expressing tumors (62, 64). Future studies may seek to determine if tailored FA blockade or simultaneously targeting of FA transporters can alter exogenous lipid uptake to enhance the sensitivity of tumor cells in lipid-rich microenvironments to virotherapy. Collectively, our work shows for the first time that fatty acid blockade can improve the therapeutic impact of OV therapy in lipid-rich TMEs.

The therapeutic advantage of FATP2 targeting during virotherapy treatment could be due to several reasons. FATP2 transports long and very long chain fatty acids but has a demonstrated preference for polyunsaturated fatty acids (PUFAs) (65). Several PUFAs, including docosahexaenoic acid and arachidonic acid, have previously been shown to have anti-viral activity by diverse mechanisms, including by interfering with binding to host entry receptors or a post-entry mechanism such as inhibiting genome replication (66, 67). Characterization of PUFA species and their relative quantity may provide clues about the lipid environment contributing to FATP2-mediated OV resistance. Further characterization of FATP2-mediated fatty acid transport and the intracellular metabolic fate of translocated fatty acids may shed more light on how FATP2 guided uptake of fatty acid species drives the OV resistance observed in our studies.

While the mechanisms of lipid-mediated virus impairment are not yet well understood, it is plausible to speculate a link between the endoplasmic reticulum (ER) stress, lipid metabolism and the antiviral state observed in cancer cells exposed to adipocyte-secreted molecules. ER is a key organelle involved synthesizing, folding, and modifying proteins. When the protein folding capacity of the ER is disrupted, increasing quantities of unfolded or misfolded proteins are detected by ER resident sensors. This leads to the activation of the unfolded protein response as several mechanisms intended to reinstate ER homeostasis. Lipid metabolism and ER stress are closely linked and bi-directional. For example, some lipid species, including fatty acids, have been shown to induce ER stress and the expression of the thioredoxin-interacting protein (TXNIP) (68). TXNIP inhibits the antioxidative effects of thioredoxin, an important regulator in redox signaling, and it is also implicated in regulating cellular metabolism and ER stress (69, 70). Our RNA sequencing data revealed TXNIP as an upregulated gene in ACM conditions (**Supplementary Figure 5B**). Given that viruses exploit the ER for replication, assembly, and egress, an impairment to ER function due to a lipotoxic load can form a significant barrier to successful infection. In fact, some evidence suggests that downregulating TXNIP may have a sensitizing effect on virus infection. In a study by Tiwarekar et al., silencing of this gene increased Measles virus replication (71). Albeit speculative, it is reasonable to hypothesize that the benefit of combinatorial treatment with the FATP2 inhibitor Lipofermata may at least partially derive from Lipofermata-mediated ER stress relief. Previous studies showed that Lipofermata-attenuated palmitate transport corresponded with a decrease in the expression of lipotoxicity mediated cell stress markers (52). Future studies may seek to determine the role of TXNIP and ER stress in lipotoxic TMEs, in the context of OV therapy. Testing combinatorial treatments that include drugs that restore ER homeostasis and virotherapy in tumors homed in adipose-rich microenvironments could provide opportunities to characterize new OV resistance mechanisms and develop new therapeutic approaches. Overall, a deeper understanding of how the adipocyte-cancer cell crosstalk influences virus-based therapies would allow the enhancement of OV therapy and unlock other potential new therapeutic avenues for tumors surrounded by environments where fat cells are abundant.

## Methods

The research presented herein complies with all ethical regulations at OHRI and the University of Ottawa (biohazardous material use certificate GC317-125-12). All animal studies were approved by the institutional animal care committee of the University of Ottawa (Protocol ID: OHRI2870) and carried out following the guidelines of the National Institutes of Health and the Canadian Council on animal care.

### Cell lines and cell culture conditions

Cell lines (OVCAR8, SKOV3, OVCAR433, MDA MB 231, BT549, MCF-7, 4T1, EMT6, EO771, B16-F10, 3T3-L1) were purchased from American Type Culture Collection (ATCC, Manassas, VA), except ID8 p53-/- which was a gift from Dr. Iain McNeish (Barts Cancer Institute, Queen Mary University of London, UK), and STOSE cells which were a gift from Dr. Barbara Vanderhyden’s lab (Ottawa Hospital Research Institute, Ottawa, ON). Cells were cultured in RPMI-1640 with 10% FBS and buffered with 25mM HEPES, except for MDA MB 231 and MCF-7, which were maintained in DMEM with 10% FBS. Vero cells were cultured in a medium containing %10 NCS/FBS mix (3:1 NCS: FBS). STOSE cells were cultured in DMEM containing 5% FBS and ITS (5μg/mL insulin, 5μg/mL transferrin and 5ng/mL sodium selenite; Gibco). Human breast and visceral preadipocytes (Zenbio) were differentiated with the appropriate proprietary adipocyte differentiation medium (Zenbio). Briefly, the preadipocytes were cultured in RPMI containing 10% FBS in T75 flasks until at least 80% confluency. The preadipocytes were cultured in the appropriate differentiation medium for ten days, with a medium change every 3-4 days. Following differentiation, the cells were acclimatized to a regular culture medium (RPMI-1640 with 10% FBS and containing HEPES) for at least five days prior to the collection of adipocyte-conditioned medium (ACM). ACM was harvested every 48-72 hours. Each ACM batch was quality controlled by conducting a test with cancer cells that are highly susceptible to oncolytic virus infection. The cells received control media or ACM and were infected with a GFP-expressing oncolytic virus. After a 24-hour period, microscopy studies were conducted to assess GFP expression, a proxy for infection, and oncolytic virus mediated cell death. For each ACM batch, we looked for resistance that was >60-70% (vs CTL media). The inhibitory effect did not vary greatly between batches. The medium was centrifuged (1500rpm, 5mins), and the supernatant was stored at -20°C until further use. Murine 3T3-L1 cells were differentiated in the recommended differentiation medium (Zenbio). All cell lines were incubated at 37°C in a 5% CO_2_ humidified incubator. All cells were tested with the e-Myco VALiD Myco PCR detection kit (FroggaBio) or Hoescht’s staining to ensure they were devoid of mycoplasma contamination prior to the experiments described.

### Generation of OVCAR8 cells over-expressing FATP2

Tetracycline inducible over-expressing cell lines were generated using lentiviral vectors. Briefly, Lenti-X™ 293T cells (Takara Bio) were transfected with packaging plasmids pMD2G and pPAX2 and a doxycycline inducible pTRIPZ lentivirus vector encoding human FATP2-FLAG tag (synthesized by GenScript). OVCAR8 cells were transduced with cell-free lentiviral vector-containing supernatant in the presence of 1 µg/mL of polybrene (Sigma, St Louis, MO, USA) and were selected with 1 μg/mL of puromycin. Human FATP2 expression was induced with doxycycline (1 μg/mL), and 48 hours post-induction, the FATP2 overexpression was demonstrated by western blot analysis using FATP2 polyclonal antibody (Proteintech, 14048-1-AP) and monoclonal Anti-FLAG^®^ M2 antibody (Millipore Sigma, F1804-200UG).

### Viruses

#### Propagation and purification

The oncolytic VSVΔ51, Maraba MG1, oncolytic HSV-1, oncolytic MeV, and VV TK^-^ VGF^-^ virus backbones and propagation and purification protocols have previously been described (24).

#### Titration of VSV and HSV infectious particles by plaque assay

The day prior, 8E5 or 2E5 Vero cells were seeded on 6-well or 12-well, respectively. Infectious viral particles from the supernatant of VSVΔ51-infected cells or virus particles from the supernatant and cell-associated viral particles from HSV-infected cells were pooled (cell-associated viruses were liberated by bursting the cells with 3 freeze-thaw cycles by freezing the sample at -80°C and thawing in a 37°C water bath until thawed but before warm). The sample was clarified of cell debris by centrifugation (1500rpm, 10mins, 4°C). The virus samples were then diluted in a 10-fold serial dilution in cold serum-free DMEM medium in deep-well plates. The medium in the plates containing the Vero cells was aspirated and replaced with 500-600μL of the serially diluted virus. The inoculated plates were incubated in the cell incubator (37°C) with gentle rocking every 10-15mins. After 45 minutes, the inoculum was aspirated and replaced with an overlay medium [1:1 mixture of 6% carboxymethyl cellulose (CMC; Sigma): DMEM (2X concentrated with 20% NCS/FBS mix)]. After a 48-hour incubation, the overlay medium was aspirated, and the cells were gently washed with PBS twice and stained with crystal violet (0.1% crystal violet in 80% MeOH in water) for 20-30 minutes. The titers were reported as plaque-forming units (PFU) per mL of sample.

#### Titration of VACV by plaque assay

Infected cells undergo three freeze-thaw cycles to release intracellular particles. Following centrifugal clarification of cell debris, the sample is tittered similarly to the plaque assay of VSV described above, with a few modifications. The samples were tittered on U2OS cells, and the overlay medium is made with 3% Carboxymethyl cellulose (CMC). The titers were reported as PFU per mL of sample.

#### MeV titration

The supernatant of MeV infected cells was serially diluted in a 10-fold serial dilution. 10μL of the diluted viral stock was combined with 90μL of Vero cells prepared in a 150 000 cells/mL solution in DMEM (10%FBS) in the wells of a 96-well plate. The plate was incubated in a 37°C incubator for three days. The titer was determined based on the 50% tissue culture infective dose endpoint method described by Spearman–Karber. The titers were reported as PFU per mL of sample.

#### Titration of tumor samples

Tumors were harvested, weighed, and transferred to 2mL Qiagen tubes containing 500uL of PBS-containing Complete™ Mini EDTA-free Protease inhibitor cocktail (1 tablet per 10mL PBS). The tumors were homogenized using a tissue lyser (TissueLyser II, Qiagen). The cell debris was spun down with a benchtop centrifuge (14000 rpm, 10 mins). The remaining cell debris was removed by passing the sample through a 70μm cell strainer (Fisherband). The sample was titered by plaque assay as described above; however, penicillin streptomycin (100 U/mL; Thermo Fisher Scientific) was added to the overlay medium. The titers were reported as PFU per milligram of tumor.

### OV pre-incubation with ACM

VSVΔ51-GFP or VACV were incubated in Eppendorf tubes containing RPMI-1640 (10% FBS, HEPES) or ACM in a 37°C incubator or at room temperature for various lengths of time. These pre-treated viral stocks were then used to infect cancer cells.

### Modified plaque assay to assess virus entry

The modified plaque assay to assess virus entry was adapted from Xu et al. Briefly, OVCAR8 cells were incubated in ACM at the indicated lengths of time before infection with 150 PFU of VSVΔ51-GFP. After an hour period of infection, the virus inoculum was removed and replaced with an overlay medium (1:1 mixture of 6% CMC: 2X DMEM with 20% NCS/FBS mix). After 48 hours, the overlay was removed, the cell monolayer was washed with PBS, and the wells were stained with crystal violet solution (0.1% crystal violet with 80% MeOH). The plates were left to air dry overnight, and the PFUs were counted the next day.

### Using VLPs to assess viral entry

Gag-GFP loaded VLPs pseudotyped with VSV-G glycoprotein were generated using a previously described protocol (72). Briefly, following transfection of plasmids encoding VLP components in HEK293T cells (ATCC), VLPs were harvested and concentrated as per manufacturer’s instructions (Lenti-X concentrator; Takara) to a 1 mL suspension in PBS. OVCAR8 cells were seeded at 80% confluency in 6-well plates and were cultured in ACM for at least 4 hours before they were transduced with 30μL of VLPs [in the presence of 0.8μg/mL polybrene (Sigma)] or infected with VSVΔ51-GFP (MOI 0.1). After 24 hours, the cells were harvested, stained with propidium iodide, and assessed by flow cytometry.

### Quantitative real-time PCR

Cell monolayers were washed twice with PBS and the total RNA was extracted using the NucleoZOL kit (Macherey-Nagel). The RNA pellets were resuspended in 20 uL of nuclease-free water. RNA quantification was achieved using the Nanodrop™ One Microvolume UV-Vis Spectrophotometer (Thermo Fisher Scientific). cDNA was generated using the iScriptTM cDNA Synthesis Kit (Bio-Rad) using 1μg of RNA. Amplification was achieved using the QuantiTect SYBR Green PCR Kit (Qiagen) in conjunction with Applied Biosystems™ 7500 Fast Real-Time PCR System analysis. Gene expression was determined relative to rPlp0 using the delta delta Ct method [2^(ΔΔCt)]. Primers were acquired from IDT (Integrated DNA Technologies), and primer optimization was determined using the standard curve method. Primer sequences are shown in **Supplemental Table 1**.

### Flow cytometry

Visualization of neutral lipids was achieved with Bodipy 493/503 (Molecular Probes) using a previously described protocol. Briefly, cells in 6-well plates were washed with PBS to remove medium and serum. They were then incubated with Bodipy staining solution (1:1000 diluted in PBS) for 15 minutes in the dark at 37°C. The cells were rinsed with PBS to remove the staining solution and trypsinized (Trypsin-EDTA 0.25%) to dislodge the cells from the well. The cells were resuspended in 5mL PBS, transferred to a 15mL conical tube, and pelleted by centrifugation (1500 rpm, 5 minutes). Next, the cells were resuspended in 200 μL of PBS and transferred to a 96-well V-bottom plate (Corning^®^ 96-well Clear V-Bottom TC-treated Microplate). After centrifugation (1500 rpm, 5 minutes) to pellet the cells, they were stained with fixable viability stain 510 (BD Horizon™; 0.5μL of stain was added per well in a total volume of 25 μL PBS) and incubated for 15 minutes in the dark. The cells were diluted with 150 μL PBS and centrifuged (1500 rpm, 5 minutes). Then, the cells were washed with flow buffer once before resuspending in 200 μL of flow buffer and transferred to 1.1 mL tall microtubes (Thermo Scientific™ Microtubes for 1.1mL Microtube System). The samples were analyzed on the BD LSR Fortessa or BD Celesta flow cytometers at the University of Ottawa Flow Cytometry and Virometry core facility (Roger Guindon Hall, University of Ottawa). Data analysis was conducted on FlowJo software (FlowJo, LLC, Ashland, OR).

### Cytotoxicity analysis

#### Crystal violet staining

The medium was aspirated, and the cells were stained with crystal violet solution (0.5% crystal violet in 80% MeOH in water) for 40 minutes on a benchtop rocker. The crystal violet was removed, and the monolayer was gently washed with tap water twice and left to air dry overnight. Qualitative analysis of the stained plates was achieved by scanning the plates. Cytotoxicity was quantified by lifting the crystal violet staining from the stained plates. The plates were incubated with 100% methanol on a shaker for 40 minutes. The methanol containing crystal violet was transferred to a 96-well plate, and the OD570 was read using the Multiskan Ascent plate reader.

#### LDH assay

A CyQuantTM cytotoxicity assay (ThermoFisher) was used as per the manufacture’s protocol to measure virus-induced cell death.

### Immunofluorescence microscopy

Brightfield and GFP filter images of cells seeded on plates were acquired with an AMG EVOS fluorescence microscope (Advanced Microscopy Group, Washington, USA). Immunofluorescence images were also obtained of cells that were grown on glass coverslips. As per a previously described protocol, lipid staining was achieved with Bodipy 493/503 (Molecular Probes). Briefly, cells were washed with PBS and incubated with a Bodipy staining solution (1:1000 in PBS; 15 minutes, 37°C) in the dark. Next, the cells were washed with PBS and fixed with 4% PFA (Thermo Fisher) for 20 minutes. The PFA was aspirated, and the cells were washed twice with PBS before the coverslips were mounted on slides with a mounting medium containing DAPI (ProLong™ Gold Antifade Mounting solution, Thermo Fisher Scientific). The slides were visualized with Zeiss Fluorescent Microscopes (ZEISS, Germany).

### Evaluating interferon signaling

#### IFNAR blocking

ACM-receiving cells incurred a medium change to ACM the evening before the day of the experiment. Anti-human IFNAR2 neutralizing monoclonal antibody (2ug; PBL Assay Science) was added to wells, and four hours later, human IFN-α 2b (200U; Sigma Aldrich) was added to the appropriate wells. Four hours post-IFN-α addition, the cells were infected with VSVΔ51-GFP (MOI 1).

#### JAK inhibition

Cell lines or ovarian patient ascites cells were cultured in ACM overnight. The next day the cells were treated with 1μM Jaki (EMD Millipore) for 3 hours before receiving 200 U/mL human IFN-α 2b (Sigma Aldrich). After 3 hours of IFN-α treatment, the cells were infected with VSVΔ51.

### Proteinase-K treatments, heat-inactivation, and boiling studies

Each sample was treated with 1U of proteinase K-linked to agarose beads (Sigma-Aldrich) for 2-4 hours at 37°C. The proteinase-K linked agarose beads were removed by pelleting *via* centrifugation (500 x g, 5 minutes). Untreated ACM or proteinase-K treated ACM were run on an SDS-PAGE gel and stained with Coomassie blue. The efficacy of protein digestion was evaluated by monitoring the density of the band corresponding to BSA. The effect of heat inactivation or boiling of ACM was evaluated by incubation of ACM in a 56°C water bath or boiling (95-100°C) on a heat block, respectively, for 30 minutes before cooling to room temperature and transferring to cells. The cells were then infected with VSVΔ51.

### LRA-mediated lipid depletion

Lipid Removal Agent (MilliporeSigma™) was mixed with PBS to achieve a working solution of 100 mg/mL. The LRA solution was added to CTL or ACM medium to reach a final LRA concentration of 4.3 mg/mL. This concentration was determined based on optimization experiments that sought to minimize cellular toxicity. CTL medium or ACM treated with LRA solution were incubated on a benchtop shaker for 30mins. Next, the samples were spun down (1500rpm, 10 minutes), and the supernatant was transferred to a new tube. The supernatant was gently drawn up while leaving a 1-2 mL buffer between the pellet and the tip of the serological pipette. Centrifugation of the supernatant was repeated twice to eliminate any LRA contaminants. Finally, the lipid-depleted medium was transferred to cells.

### Fatty acid quantification

Quantification of fatty acids in the ACM was achieved using the Free Fatty Acid Quantification Colorimetric/Fluorometric Kit (BioVision) as per the manufacture’s protocol.

### Fatty acid supplementation

Sodium palmitate (Sigma-Aldrich) was combined with heated 100% ethanol and vortexed for 15 seconds. The fatty acid solution was then heated for 5-10 minutes at 65°C with periodic vortexing. The suspension was combined with an equal volume of heated sterile water and vortexed immediately. The fatty acid solution was stored at -20°C until use. A 2% BSA-containing medium solution was prepared by adding BSA (fatty acid-free BSA; Sigma-Aldrich) to a serum-free RPMI medium. The sample was briefly mixed by swirling and warmed in a 37°C water bath to facilitate the dissolving of the BSA. The BSA-containing medium was filtered with a 0.22 μM syringe filter. The stock of sodium-palmitate in 50% ethanol was warmed on a heat block at 65°C until the fatty acid was in solution. While still warm, the sodium-fatty acid solution was added to the 2% BSA- containing medium in a dropwise fashion, immediately capped and vortexed. The palmitate supplemented medium was incubated in a 37°C water bath for three hours. The prepared fatty acid-BSA medium was supplemented with 10% FBS right before being added to cells. Cytotoxicity quantification of lifted crystal violet or OV titers were normalized to samples containing equivalent volume of 2% BSA-containing medium.

### Lipid mixture supplementation

A chemically defined lipid mixture (Sigma-Aldrich) containing non-animal derived fatty acids (2 μg/ml arachidonic and 10 μg/ml each linoleic, linolenic, myristic, oleic, palmitic, and stearic) and 0.22 mg/ml cholesterol, was diluted in RPMI medium containing 10% FBS to final concentrations of 1, 5, 10, 25, 50, 75 and 100 mL/L and added to OVCAR8 cells. Cells were pre-incubated with the lipid mixture for 1 hour before infecting with VSVΔ51-EGFP at MOI 0.1. After 48 hours, infectivity was assessed by quantifying the mean fluorescent intensity (MFI) of EGFP (excitation 488 nm, emission 510 nm) using the BioTek Synergy™ Mx Microplate Reader. Infectivity quantification was plotted as percent MFI-EGFP and normalized to samples receiving no lipid mixture. Cell viability was also assessed with the REDOX indicator resazurin (Sigma Aldrich) according to the manufacturer’s protocol. Fluorescence was measured (excitation 530 nm, emission 590 nm) using the BioTek Synergy™ Mx Microplate Reader. Cell viability (metabolism) was plotted as percent viability and normalized to samples receiving a 50% lipid mixture. Cytotoxicity was assessed using a crystal violet assay. The medium was removed from wells, and cells were washed once with PBS before 0.5% crystal violet (80% MeOH in water) was added to each well. Plates were incubated at room temperature on a shaker for 20 minutes, then crystal violet solution was removed, and fixed cells were washed three times with water. Plates were left to air dry overnight; once dry, plates were scanned.

### Assessment of mitochondrial respiration using seahorse technology

Oligomycin (1.5 μM), trifluoromethoxy carbonylcyanide phenylhydrazone (FCCP) (0.5 μM), antimycin, and rotenone (0.5 μM) treatment were injected at designated intervals as per the manufacturer’s instructions for Seahorse XF Cell Mito Stress Test Kit (Agilent). The effect of acute etomoxir (Cayman Chemical Company) treatment was evaluated by injection of the drug after three baseline readings.

### siRNA knockdown of genes involved in FA transport

siRNAs [SMARTpool ON-TARGETplus Non-targeting Control Pool, SMARTpool ON-TARGETplus Human SLC27A1, SMARTpool ON-TARGETplus Human SLC27A2, SMARTpool ON-TARGETplus Human SLC27A4)] were purchased from Dharmacon and resuspended in siRNA Buffer (5X siRNA Buffer, Dharmacon) to generate 20 μM stocks. For transfection, 5 μL of Lipofectamine™ RNAiMAX Transfection Reagent (Thermo Fisher Scientific) was combined with 250 μL Opti-MEM and incubated for 5 minutes. The RNAiMAX complex was then combined with siRNAs diluted in Opti-MEM (2 μL of siRNA with 250 μL of Opti-MEM) in a gentle dropwise fashion. The siRNA-RNAiMAX complex was then incubated for 20 minutes at room temperature and added to wells with 5E5 cells suspended in 1.5 mL RMPI-1640 (containing 10% FBS). 18-24 hours post-transfection, designated wells were infected with VSVΔ51-GFP for 40 minutes. The virus inoculum-containing medium was removed and replaced with a CTL medium or ACM (50% v/v).

### Lipid modulating drugs

#### TOFA

Cells were treated with 0.1, 0.5, 1, 2, 5, 8 or 10μM of 5-tetradecyloxy-2-furoic (TOFA, Millipore Sigma) for 3hours. The medium was then changed to CTL medium or ACM medium containing half the previous concentration of TOFA in the well. Two hours later, the cells were infected with VSVΔ51 (MOI 0.1).

#### Lipofermata

Cells were infected with VSVΔ51 for 40 minutes. The virus inoculum-containing medium was removed and replaced with CTL medium containing Lipofermata (Cayman Chemical Company) at the indicated concentration for 2 hours before an equivalent volume of CTL medium or ACM was added to the wells. For *in vivo* studies, Lipofermata was prepared from a 25mg/mL stock (in DMSO) to its working concentrating with dilution with DMSO alone or DMSO and 30% v/v of Kolliphor^®^EL (Sigma Aldrich). The target injection volume was 25 uL to minimize DMSO toxicity to receiving mice.

### Hematoxylin and eosin staining

Tumors were fixed in formalin as per standard protocols (24) and were stained with hematoxylin and eosin by the Pathology Department at the Ottawa Hospital.

### RNA sequencing analysis

OVCAR8 cells were cultured in CTL medium or ACM for 18 hours or cultured in CTL medium or ACM. The wells were washed with sterile PBS and the RNA was extracted (NucleoZOL kit; Macherey-Nagel). Tidyverse, EdgeR, and Heatmaply were used for bioinformatics analysis and heatmap generation.

### Animal studies and tumor models

All animal studies complied with ethical regulations and were approved by the Institutional Animal Care Committee of the University of Ottawa (animal protocol # OHRI2870) and carried out in accordance with guidelines of the National Institutes of Health and the Canadian Council on Animal Care. Balb/c or C57BL/6 or nude CD-1 (6 to 8 weeks old) female mice were acquired from Charles River Laboratories. Mice with palpable tumors were monitored daily. Mice were euthanized at the indicated experimental time point. Otherwise, animals were euthanized at the pre-established human endpoint criteria. Animals displaying signs of pain, lethargy, labored breathing, lack of responsiveness, significant abdominal distension due to ascites build up, or when tumor volume reached 1500 mm^3^, were sacrificed. Animals were blindly randomized to treatment groups upon tumor implantation and before treatments. Animals that did not develop palpable tumors were excluded from the study. All animal manipulations were conducted with the operator blinded to the experimental condition and allocation group. Tumor volume was calculated using the following formula: tumor volume = 1/2(length × width^2^). HFD (TD.06414) or a RD control (T. 08806) fed C57BL/6 mice received a specialized diet from Harlan Laboratories (Teklad Custom Diet; Envigo) for 8-10 weeks until a substantial change in the average mouse weight was observed (at least 20-25%). The mice remained on the corresponding diet until the end of the experiment. Cells for implantation were cultured until 60-70% confluency. The cells were washed twice with PBS and passed through a 70 μM cell strainer. 2E5 cells (EMT6, 4T1), 5E5 cells (EO771) or 5E6 cells (ID8-F3 p53-/-, OVCAR8) resuspended in sterile PBS were implanted SC/FP or IP, respectively. Tumor models in mice were generated by implanting syngeneic tumor cells in the FP, IP, or subcutaneously. Intrabursal STOSE models were generated by surgical implantation as previously described (38, 39).

### Statistical analysis

Statistical significance was determined using an unpaired t-test, one-way or two-way analysis of variance (ANOVA) as indicated in the figure legend. Log-rank Mantel-Cox test was used to assess survival. The number of biological replicates and the statistical test used are indicated in the figure legends. Error bars represent the standard error of mean or standard deviation as indicated. P-values less than 0.05 were deemed significant. If no indication is shown, the results are non-significant. Statistical analyses were performed using GraphPad Prism 9 software. The statistical significance of all *p*-values is: ^ns^ *p*>0.05, *p<0.05, **p<0.01, ***p<0.001 and ****p<0.0001. Whenever possible exact p-values are provided in the text, figure legends or figures.

## Data availability statement

The data presented in this study are deposited in the NCBI (BioProject) repository, accession number PRJNA937284 (https://www.ncbi.nlm.nih.gov/bioproject/PRJNA937284).

## Ethics statement

The animal study was reviewed and approved by Animal Care Committee of the University of Ottawa.

## Author contributions

AS, MJ, JP, RB, MC, CL, JD JM, VT, RR, EF, HB, CT, SN, BW, ST, TA, and CI conducted *in vitro* experiments. AS, MJ, JPo, JPe, CT, NM, JB, BA, NC, NB, and CI. performed mouse experiments. AS, MJ, JP, RB, MC, and CI wrote the manuscript. AS, BV, L-HT, JB, and CI contributed to design of studies. All authors contributed to the article and approved the submitted version.

## Supplementary material

Supplementary Figure 1 Adipocyte-secreted factors impairs oncolytic virus infection in a dose-dependent fashion. **(A)** Body weights of C57bl/6 mice fed with regular chow (RD) or a high-fat diet (HFD). **(B)** Following a period of high-fat or regular diet feeding of C57BL/6 mice, EO771 cells were seeded in the FP. When tumors reach ~ 1 cm^3^, they were collected, fixed, and stained with hematoxylin and eosin. Scale bar represents 400 μm. **(C)** Representative images of the breast and mouse 3T3-L1 preadipocyte and mature adipocyte cultures after a 10-day differentiation protocol. **(D)** OVCAR8 cells were cultured in the indicated medium for 24 hours, and the total number of viable cells was counted with a ViCell Analyzer. Data indicate the mean ± SD of 3 biological replicates. One-way ANOVA, Dunnett’s multiple comparisons test, showed non-significant differences among groups. **(E)** SKOV3 cells were cultured in CTL medium, preadipocyte-conditioned medium (Pre-ACM), or ACM overnight before being infected with VSVΔ51 (MOI 0.1) for 48 hours. Supernatants were collected and released infectious particles were quantified by plaque assay. Data indicate the mean ± SEM of 3 biological replicates. Two-way ANOVA. **(F)** The indicated cell lines were cultured in increasing concentrations of ACM overnight before being infected with VSVΔ51 (MOI 0.1) for 48 hours. Cell monolayers were stained with crystal violet for cytotoxicity analysis. **(G)** Representative images of indicated cancer cell lines cultured in CTL medium or ACM overnight before being infected with VSVΔ51-eGFP (MOI 0.1) for 48 hours.

Supplementary Figure 2 ACM-mediated OV resistance can be induced by various adipocyte types and is conserved across human and mouse species. **(A)** Representative images from three independent experiments of ascites-derived ovarian cancer cells (AF2068) cultured in breast ACM or visceral ACM (VACM) and infected with GFP-expressing oncolytic Maraba MG1 (MOI 0.1, 48 hpi) or VACV (MOI 0.1, 48 hpi). Scale bar 20 µm. **(B,C)** A small panel of human breast or ovarian cancer cell lines were cultured in visceral ACM and infected with VSVΔ51 (B) or VACV (C). All infections were done at MOI 0.1 except for BT549 cells that were infected with VACV MOI 1, and infectious particles were quantified after 48 hours of infection. Data indicate the mean ± SEM (n=3). **(D)** Mouse cancer cells were cultured in human ACM and infected with VSVΔ51 [MOI 0.1 (E0771 and B16-F10) or MOI 1 (4T1 and EMT6)] and infectious particles were quantified and plotted after 48 hpi. **(E,F)** OV titers of human breast or ovarian cancer cell lines cultured with ACM from murine adipocytes, 3T3-L1, and infected with VSVΔ51 [(E), MOI 0.01, except for OVCAR433 which was infected at MOI 0.1] or VACV [(F), MOI 0.01)]. Data indicate the mean ± SEM (n=3 to 5). Two-tailed, unpaired t-test. *p <0.05, **p <0.01, ***p <0.001, ****p<0.0001.

Supplementary Figure 3 Adipocyte-secreted factors do not impede VSV-G- mediated cell entry. **(A, B)** Representative images (A) or flow cytometry plots (B) of OVCAR8 cells cultured overnight in CTL medium or ACM and transduced with GFP-loaded VLPs pseudotyped with VSV-G glycoprotein or infected with VSVΔ51-eGFP (MOI 0.1) for 24 hours (n=4). **(C)** The gating strategy for flow cytometry experiments is shown using an unstained sample as an example.

Supplementary Figure 4 ACM-mediated OV resistance is not induced by Type I interferons or a protease-sensitive and thermolabile polypeptide. **(A)** OVCAR8 cells incubated overnight in CTL medium, or ACM were treated with IFN-α (200U) or IFNAR blocking antibody (2μg) or both before infection with GFP-expressing VSVΔ51 (MOI 1, 48 hours). Scale bar represents 1000 μm. **(B)** Proteinase-K(PK)-treated CTL medium or ACM were resolved on an SDS-PAGE gel and stained with Coomassie blue. **(C-E)** PK -treated CTL or ACM medium was added to OVCAR8 cells and infected with the indicated OV platform (MOI 0.1, 48). Representative images are shown in panel C, scale bar= 1000 μm. Infectious particles were quantified by plaque assay (D,E). Data indicate the mean ± SEM (n=3 to 6). **(F-G)** CTL medium or ACM were heat-inactivated by boiled (F) or incubation in a 56°C water bath (G) for 30 mins. After cooling to room temperature, the medium was added to OVCAR8 cells and infected with VSVΔ51-GFP (MOI 1, 24pi) or SKOV3 cells and infected with VSVΔ51 (MOI 0.1, 48 hpi). Representative images of OVCAR8 cells are shown in F (scale bar= 1000 μm), and quantification of infectious particles produced by SKOV3 cells is plotted in panel G, respectively. Data indicate the mean ± SEM (n=3). Two-way ANOVA, Tukey’s multiple comparison test. *p <0.05.

Supplementary Figure 5 Adipocytes secrete fatty acids and alter the expression of lipid metabolic enzymes in cancer cells. **(A)** The media (CTL and ACM) used to culture cancer cells were assessed for fatty acid content using a commercially available quantification kit. Data indicate the mean ± SEM (n=3). Two-tailed, unpaired t-test. **(B)** OVCAR8 cells were cultured in CTL medium or ACM for 18 hours before RNA was extracted for transcriptomic analysis. The heatmap denotes 99 differentially expressed significantly altered genes (p<0.05) scaled by z-score. Two biological replicates (R1 and R2) are shown for each experimental condition. Heatmap was generated using Heatmaply. **(C,D)** Two human ovarian cancer cell lines were cultured in ACM for 24 hours (SKOV3) or 48 hours (OVCAR8). *CPT1* transcripts were quantified by qPCR (fold change relative to *Rplp0*). Data indicate the mean ± SEM of 3 biological replicates Two tailed, unpaired t-test.

Supplementary Figure 6 Fatty acid supplementation recapitulates the anti-viral effects of ACM. **(A, B)** OVCAR8 cells in CTL medium or ACM were dosed with increasing concentrations of 5-tetradecyl-oxy-2-furoic acid (TOFA) and infected with VSVΔ51 (MOI 0.1, 48 hours). OV-mediated cell death was evaluated by crystal violet cytotoxicity assay (A), and viral titer was assessed by plaque assay (B). Data indicate the mean ± SEM of 3 biological replicates. Two-way ANOVA, Tukey’s multiple comparison test, ****p<0.0001. **(C)** CTL medium or ACM was depleted of lipids, using a lipid Removal Agent (LRA), before transferring to SKOV3 cells. After 16 hours, the intracellular lipid accumulation was assessed by flow cytometry of Bodipy stained cells. Two-tailed, unpaired t-test, *p<0.05 **(D)** Representative images of OVCAR8 cells treated with either CTL medium, ACM or medium supplemented with a chemically defined lipid mixture (FA mix) and stained Bodipy (lipids) and DAPI (nucleus). **(E)** Cytotoxicity assay was performed on cells receiving increasing concentrations of a palmitate supplemented medium and infected with VACV (MOI 3, 48 hpi) or VSVΔ51 cells (MOI 3, 24 hpi). **(F)** The effect of a lipid mixture (FA Mix) on cell viability in the absence of infection was quantified using resazurin assay and represented as percent viability relative to samples receiving no FA mixture. Data indicate the mean ± SEM (n=5). **(G-I)** Representative crystal violet staining (G) of OVCAR8 cells receiving increasing concentrations (0-25%) of lipid mixture and infected with VSVΔ51-GFP cells (MOI 0.1, 48 hpi). The effects of the FA mixture viral infectivity and cell viability were quantified using the GFP intensity (H) and a resazurin assay (I), respectively. Values are presented as percentage MFI GFP relative to samples receiving no FA mix (H) and percent viability relative to samples receiving a 50% FA mixture (I). Data indicate the mean ± SEM of 3 independent experiments. Two-way ANOVA. **p < 0.01. ***p <0.001, ****p < 0.0001.

Supplementary Figure 7 Evaluating the effect of targeting transporters of fatty acids. **(A)** OVCAR8 cells were transfected with siRNAs targeting the indicated gene transcripts. Total RNA was extracted 48 hours post-transfection to assess for target gene expression by qPCR. Fold change to relative Rplp0 loading control is plotted. Two-tailed, unpaired t-test, *p <0.05, **p <0.01. **(B)** Representative microscopy images of OVCAR8 cells were transfected with the indicated siRNAs for 16 hours and infected with VSVΔ51 (MOI 0.05). After an hour, the virus inoculum-containing medium was removed and replaced with CTL medium or ACM. **(C)** Representatives immunoblot analysis of inducible OVCAR8-FATP2 overexpressing cells. Overexpression of FLAG-tagged FATP2 in OVCAR8 cells dosed with doxycycline (DOX) is shown with anti-FATP2 and anti-FLAG antibodies. β-Actin is included as a loading control. **(D,E)** OVCAR8 were treated with lipofermata (0.24μM) and cultured in CTL medium or ACM prior were mock-treated (D) or infected with GFP-expressing VSVΔ51 [(E), MOI 0.1]. Representative images were taken 48 hours post-treatment. Scale bar represents 1000 μm.
