# Supplementary material for: Fatty acid transport protein inhibition sensitizes breast and ovarian cancers to oncolytic virus therapy via lipid modulation of the tumor microenvironment
Source: Front Immunol. 2023 Mar 10;14:1099459. doi: 10.3389/fimmu.2023.1099459 (PMC10036842; doi:10.3389/fimmu.2023.1099459)
Supplement: Supplementary Figure 1 — Adipocyte-secreted factors impairs oncolytic virus infection in a dose-dependent fashion. (A) Body weights of C57bl/6 mice fed with regular chow (RD) or a high-fat diet (HFD). (B) Following a period of high-fat or regular diet feeding of C57BL/6 mice, EO771 cells were seeded in the FP. When tumors reach ~ 1 cm3, they were collected, fixed, and stained with hematoxylin and eosin. Scale bar represents 400 μm. (C) Representative images of the breast and mouse 3T3-L1 preadipocyte and mature adipocyte cultures after a 10-day differentiation protocol. (D) OVCAR8 cells were cultured in the indicated medium for 24 hours, and the total number of viable cells was counted with a ViCell Analyzer. Data indicate the mean ± SD of 3 biological replicates. One-way ANOVA, Dunnett’s multiple comparisons test, showed non-significant differences among groups. (E) SKOV3 cells were cultured in CTL medium, preadipocyte-conditioned medium (Pre-ACM), or ACM overnight before being infected with VSVΔ51 (MOI 0.1) for 48 hours. Supernatants were collected and released infectious particles were quantified by plaque assay. Data indicate the mean ± SEM of 3 biological replicates. Two-way ANOVA. (F) The indicated cell lines were cultured in increasing concentrations of ACM overnight before being infected with VSVΔ51 (MOI 0.1) for 48 hours. Cell monolayers were stained with crystal violet for cytotoxicity analysis. (G) Representative images of indicated cancer cell lines cultured in CTL medium or ACM overnight before being infected with VSVΔ51-eGFP (MOI 0.1) for 48 hours. [file DataSheet_1.pdf]

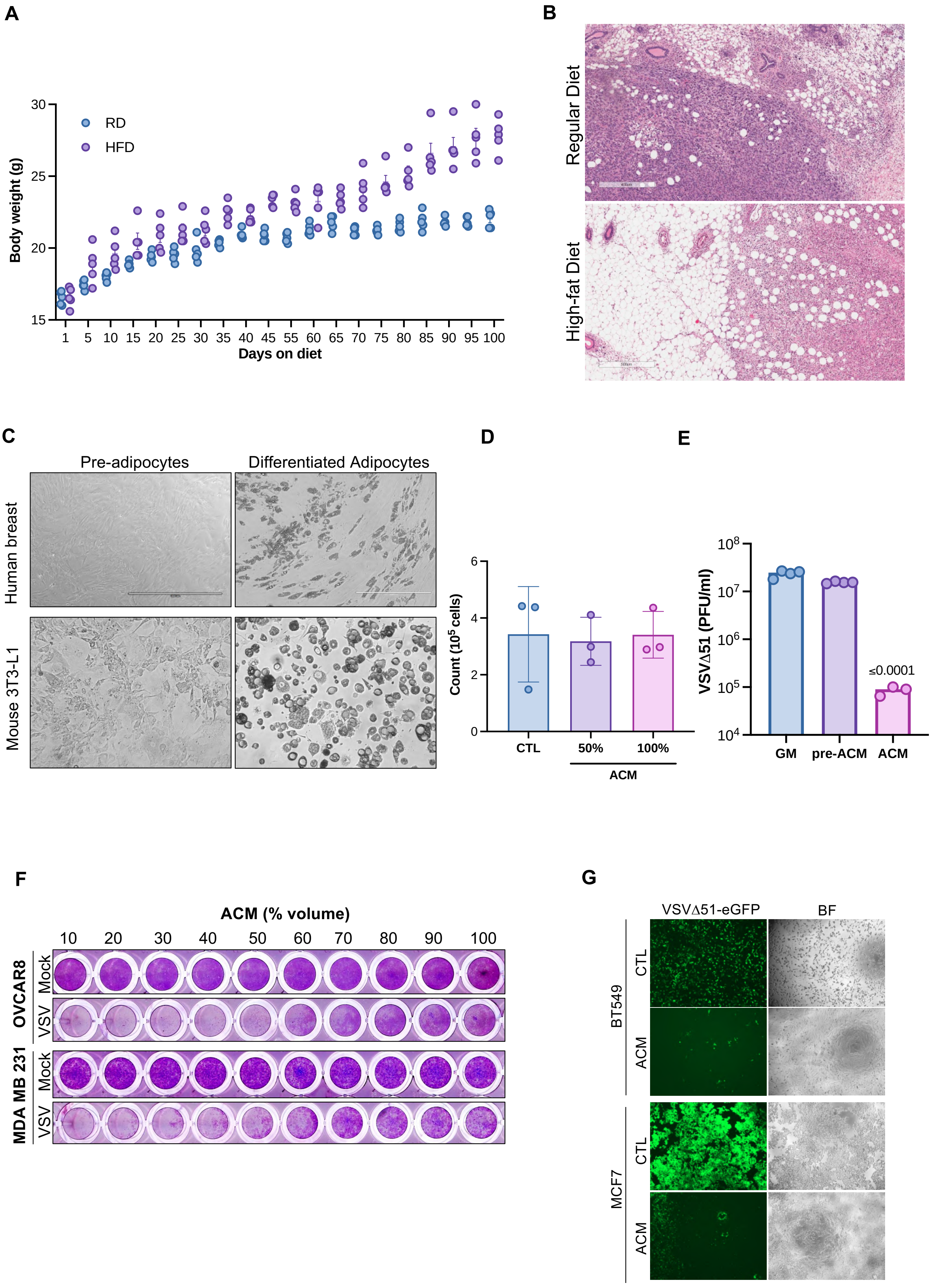

Supplementary Figure 1

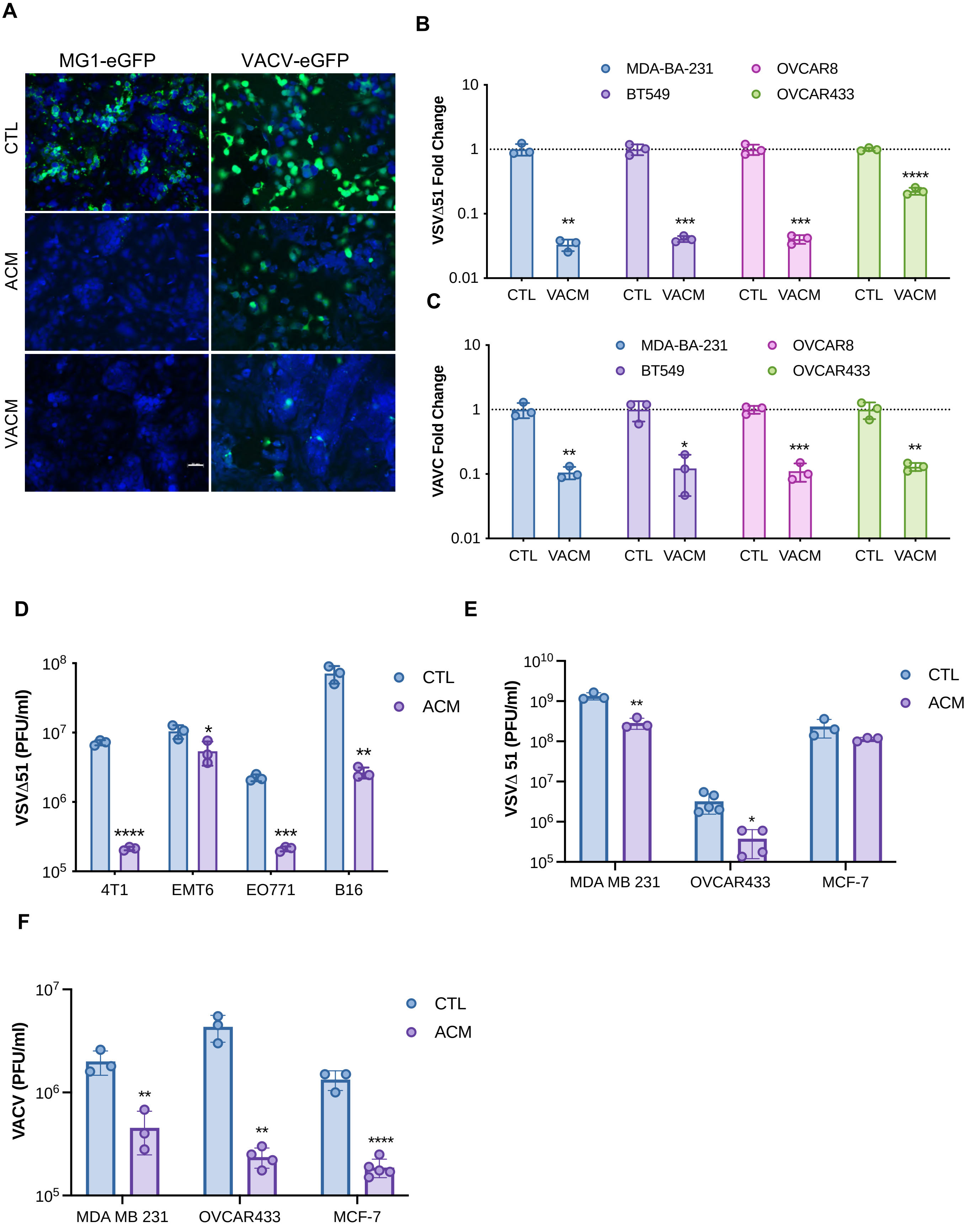

Supplementary Figure 2

A

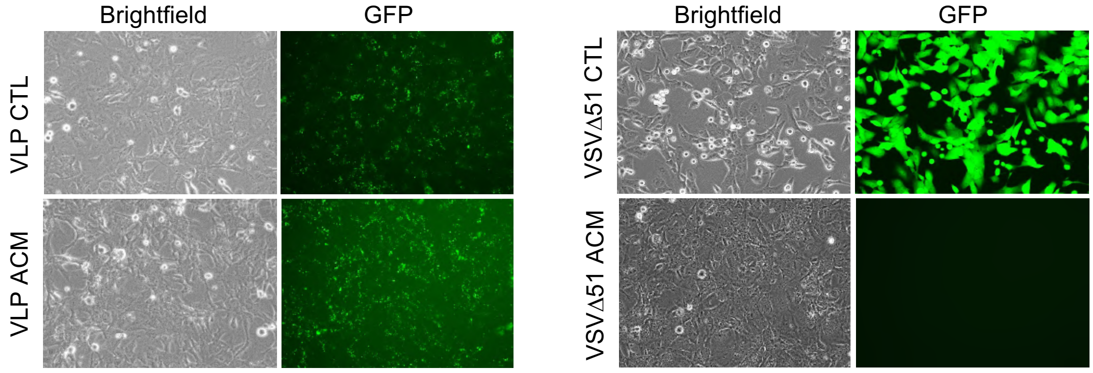

B

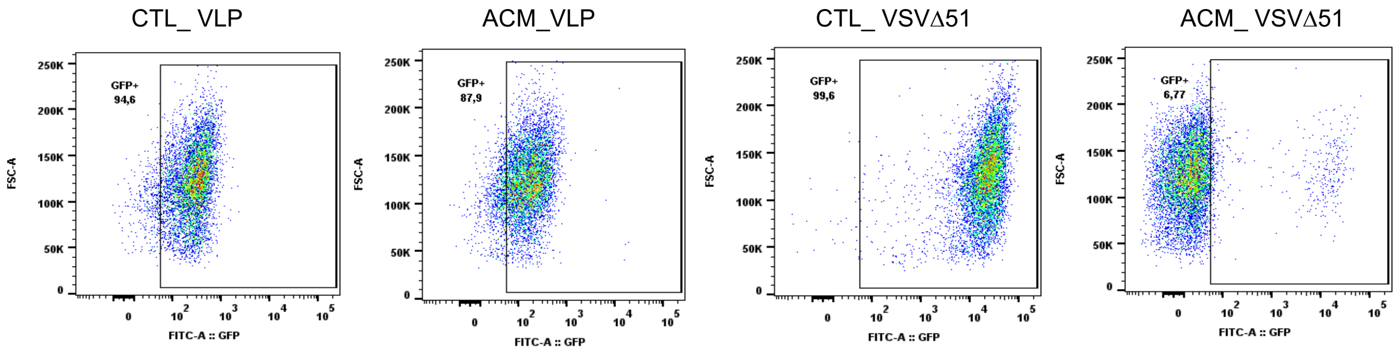

C

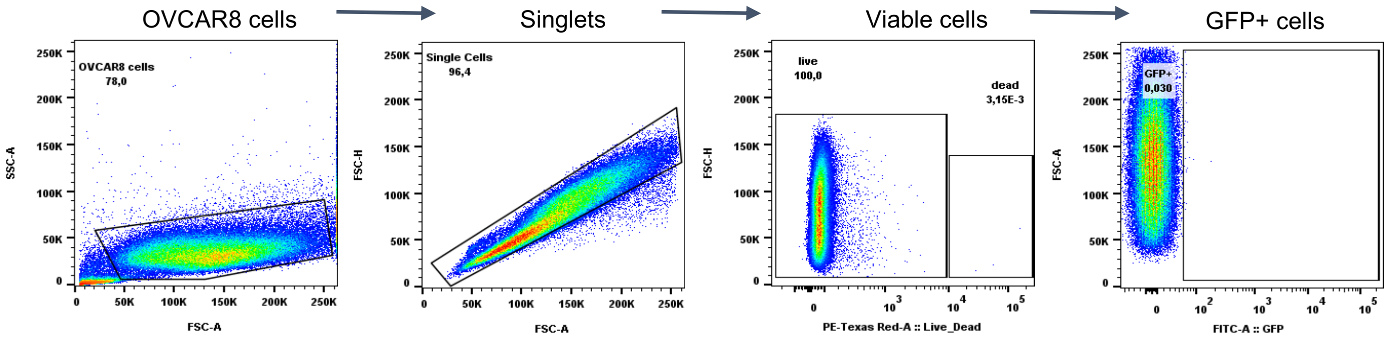

Supplementary Figure 3

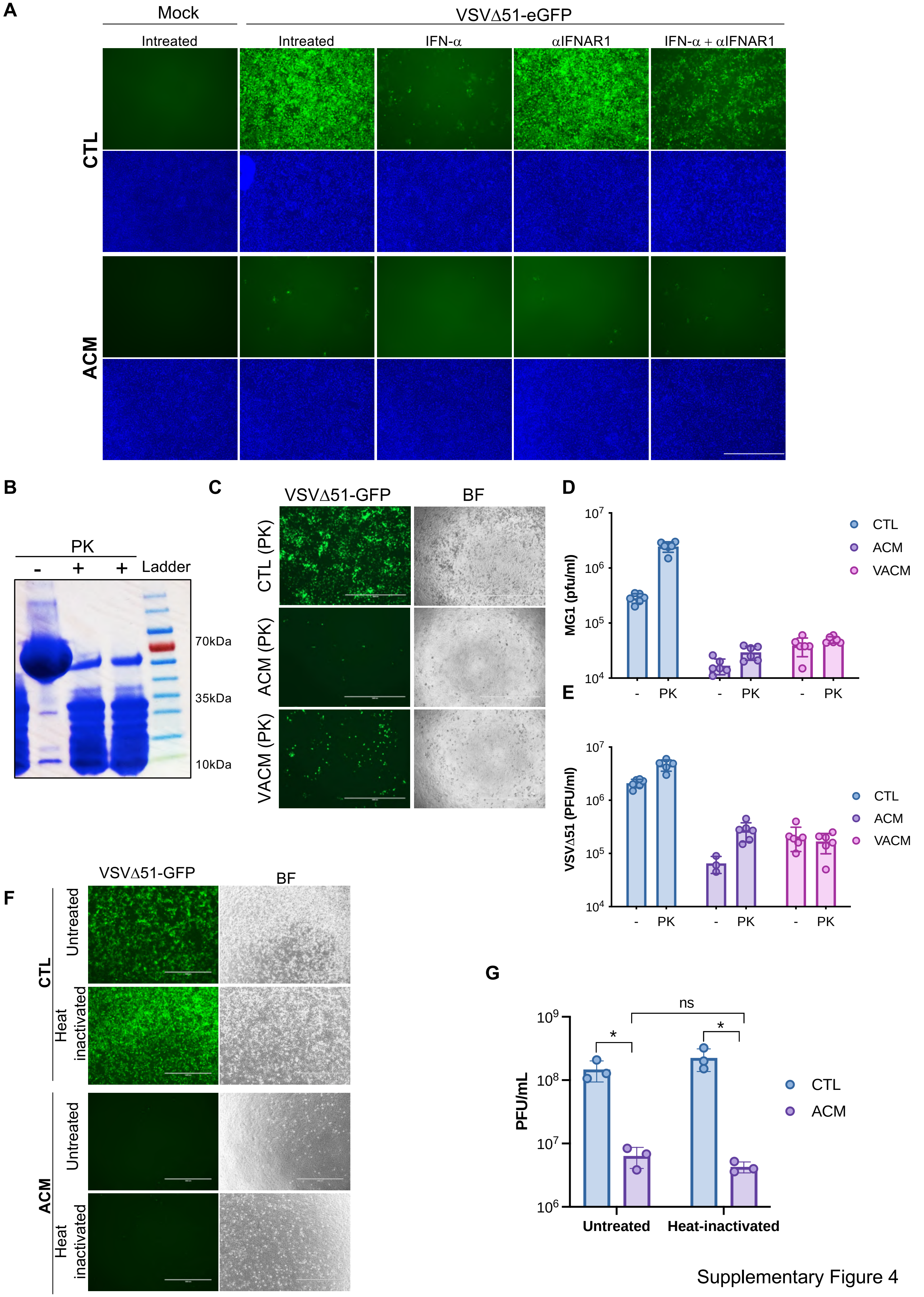

Supplementary Figure 4

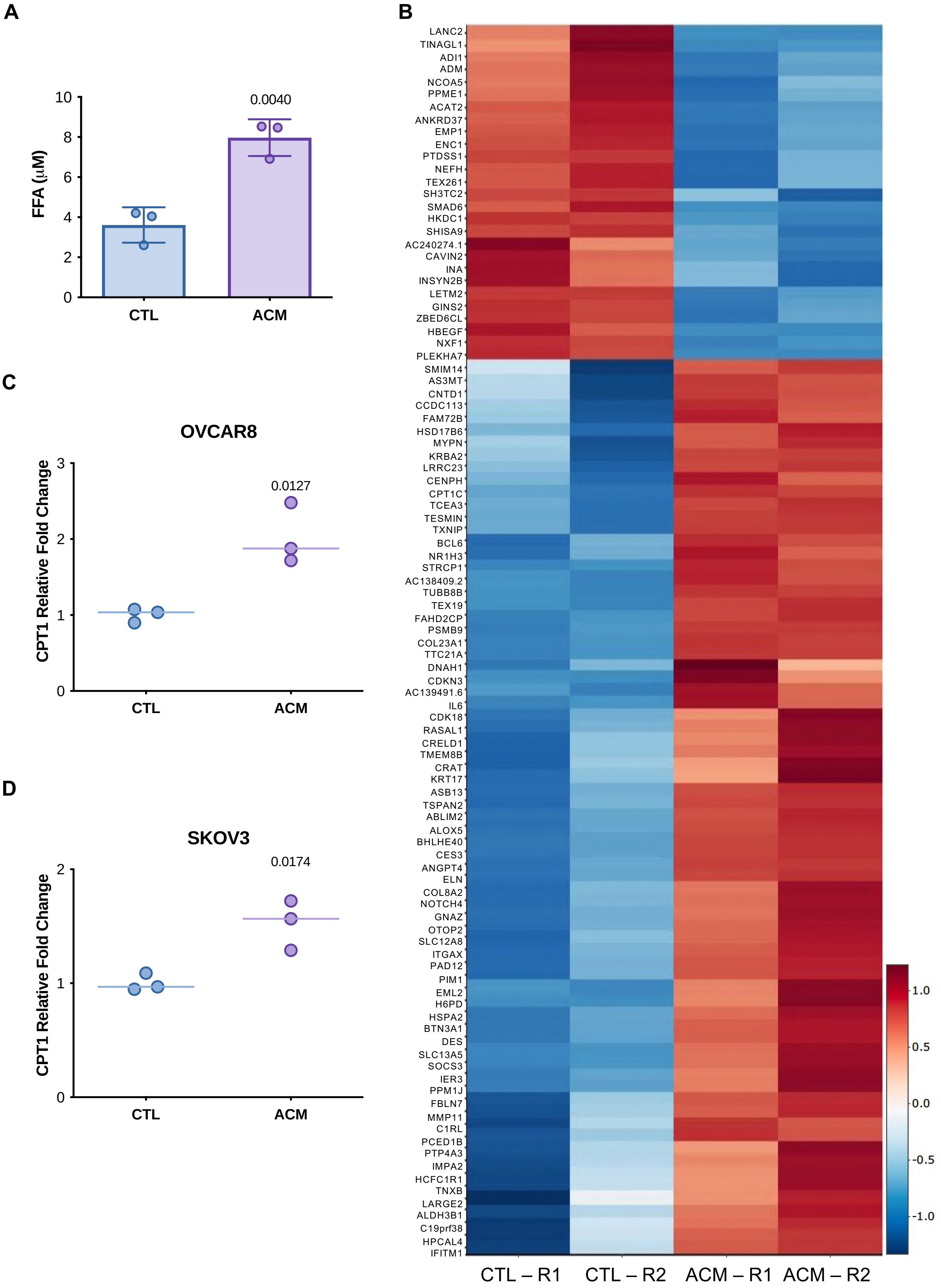

Supplementary Figure 5

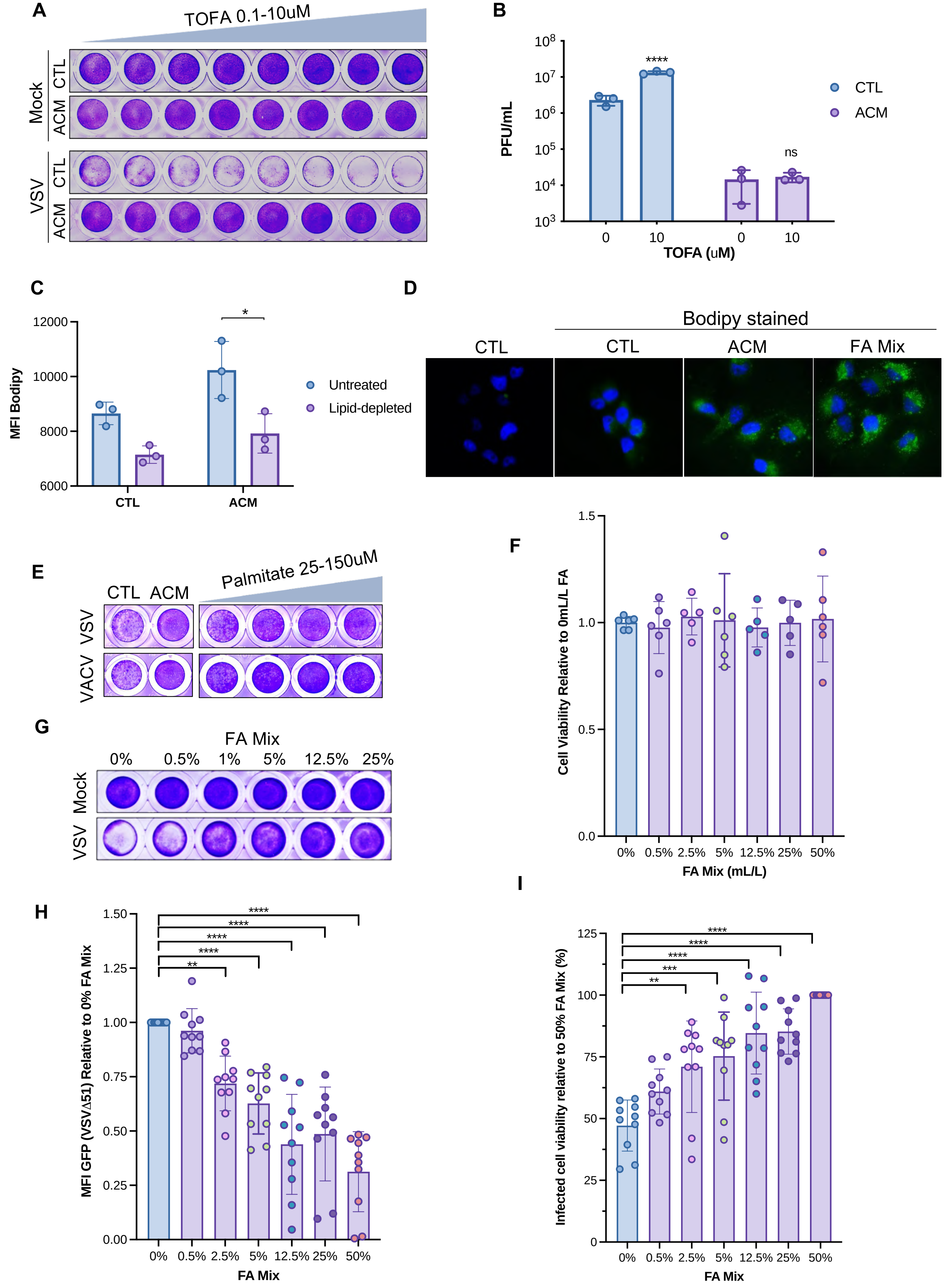

Supplementary Figure 6

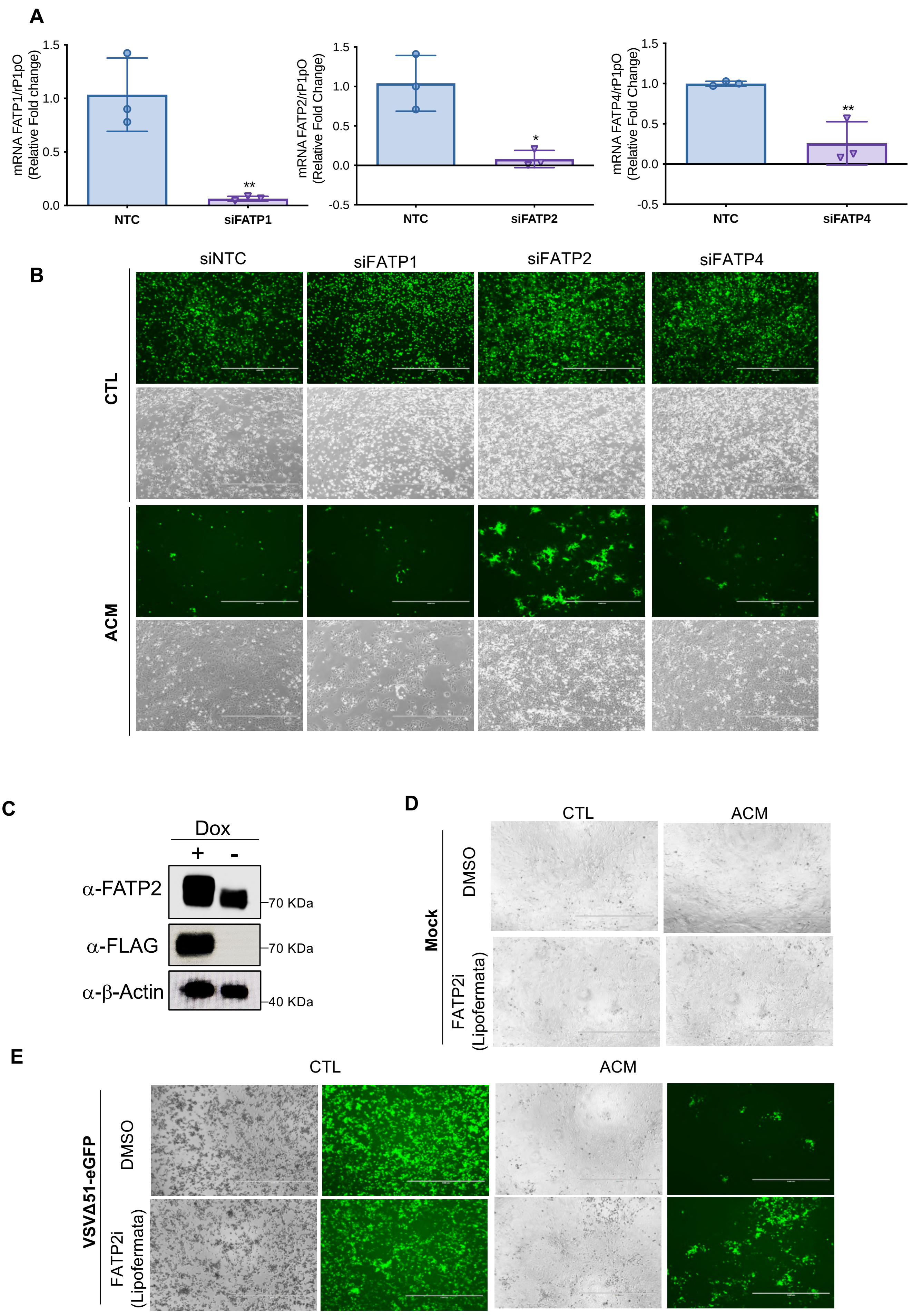

Supplementary Figure 7
