# Supplementary material for: Fatty acid transport protein inhibition sensitizes breast and ovarian cancers to oncolytic virus therapy via lipid modulation of the tumor microenvironment
Source: Front Immunol. 2023 Mar 10;14:1099459. doi: 10.3389/fimmu.2023.1099459 (PMC10036842; doi:10.3389/fimmu.2023.1099459)
Supplement: Supplementary file 2 [file DataSheet_2.docx]

**SUPPLEMENTARY TABLE 1: RT-qPCRs Primer Sequences**

| **Gene** | **Forward Sequence (5’- 3’)** | **Reverse Sequence (5’- 3’)** |
| --- | --- | --- |
| MG1-L | GGCAAGAATCGTTCTTCAGC | TCGAAGCATTTGATGAGTGG |
| DDX58 | TGTGGGCAATGTCATCAAAA | GAAGCACTTGCTACCTCTTGC |
| IFITM1 | ACTCCGTGAAGTCTAGGGACA | GACCATAAGCCGAGACACTGT |
| STAT1 | TGTATGCCATCCTCGAGAGC | AGACATCCTGCCACCTTGTG |
| OAS1 | AAGGTGGTAAAGGGTGGCTC | GCTGTCTCCTAATTTCCTGG |
| CPT1a | CCTCCAGTTGGCTTATCGTG | TTCTTCGTCTGGCTGGACAT |
| FATP1 | GCATCTGGGGAAAAGTTTGA | TGAGCCGTCCTTTGTAGTAG |
| FATP2 | TGTCGCCAGAACTACAAGCA | TGAGAACGGAACGCCTGATT |
| FATP4 | CCAGGCCTACCTTACTGGTG | GACTCGACGTGTTTTGTCCT |
| CD36 | CGGA ACTGTGGGCTCAT | GGTCTCCAACTGGCATTAGAA |
| RPLP0 | TTAAACCCTGCGTGGCAATCC | TTAAACCCTGCGTGGCAATCC |

*All are human target genes (except MG1-L)
